# Unveiling Novel Genetic Loci and Superior Alleles for Nickel Accumulation in Wheat via Genome-Wide Association Study

**DOI:** 10.3390/plants14081262

**Published:** 2025-04-21

**Authors:** Xia Shi, Shenghui Geng, Jinna Hou, Taotao Shi, Maomao Qin, Wenxu Li, Ziju Dai, Zhengfu Zhou, Minghui Zhang, Zhensheng Lei

**Affiliations:** 1 Henan Institute of Crop Molecular Breeding, Henan Academy of Agricultural Sciences, Henan Key Laboratory of Wheat Biology, National Engineering Laboratory of Wheat Key Laboratory of Wheat Biology and Genetic Breeding in Central Huanghuai Area, Ministry of Agriculture, Zhengzhou 450002, China; mrshi0614@126.com (X.S.); gengshenghui@hnagri.org.cn (S.G.); houjinna_1982@163.com (J.H.); shitao930927@163.com (T.S.); qmm1988630@126.com (M.Q.); tinbingye@163.com (W.L.); zijudai@163.com (Z.D.); zhouzf215@hnagri.org.cn (Z.Z.); 2 Nanyang Academy of Sciences, Nanyang 473000, China

**Keywords:** hexaploid wheat (*Triticum aestivum* L.), Ni accumulation, GWAS, superior and inferior alleles

## Abstract

Nickel (Ni) pollution poses significant threats to human health and crop development through the food chain. This study aimed to identify the novel genomic regions and superior alleles associated with Ni accumulation in wheat (*Triticum aestivum* L.) grains using genome-wide association analysis (GWAS) with a diversity panel of 207 bread wheat varieties. In total, five unique genetic loci associated with Ni accumulation were identified and they explained, on average, 8.20–11.29% of the phenotypic variation. Among them, three unique genetic loci were mutually verified by different statistical models in at least two environments, indicating their stability across different environments. Moreover, the highest effect quantitative trait nucleotide (QTN) AX-111126872 with a quantitative trait locus (QTL) hotspot on chromosome 6B identified in this study was not reported previously. Three putative candidate genes linked to Ni accumulation were revealed from the stable genetic loci. Among them, one gene associated with the stable genetic locus on chromosome 6B (AX-111126872) encodes the *glycine-rich proteins* (*GRPs*) as a critical factor influencing Ni accumulation in wheat grains. This study increases our understanding of the genetic architecture of Ni accumulation in wheat grains, which is potentially helpful for breeding wheat varieties without Ni toxicity.

## 1. Introduction

Nickel (Ni) pollution in soil has become a serious global environmental issue, primarily due to human activities such as metal processing, mining, and the use of Ni-containing products, which are the main causes of soil Ni pollution [1,2]. Once Ni enters the soil, they are absorbed by plants and enter the food chain, thereby affecting human health. Studies have shown that high concentrations of Ni can be carcinogenic, and long-term exposure or consumption increases the risk of cardiovascular disease, lung diseases, and various cancers [3,4]. However, Ni is also an essential trace nutrient for plants, participating in their growth and development. For example, Ni is a crucial component of plant bio-active enzymes (such as urease, aldolase, or superoxide dismutase), where urease and aldolase are involved in glutathione regeneration, reactive oxygen species scavenging, cellular defense, and redox balance, playing important roles in plant growth and development [5,6,7]. Moreover, high concentrations of Ni can lead to toxicity, inhibiting key physiological processes such as seed germination, nutrient absorption, and photosynthesis, resulting in reduced crop yields and the accumulation of Ni within crops, posing potential threats to human health and crop growth and development [8,9]. In summary, Ni pollution caused by human activities not only affects plant growth, but it also may also pose a serious threat to human health through the consumption of contaminated food crops.

Given the potential threat of Ni to plant growth and human health, a deep analysis of the genetic mechanisms underlying the accumulation characteristics and tolerance mechanisms of Ni in plants is crucial for reducing their toxic effects. Previous studies have identified several genes and genetic loci that can regulate the absorption and transport processes of Ni in plants. The *AtHAN* (high-affinity nickel/cobalt transporter) gene in *Arabidopsis thaliana* encodes Ni with high affinity for both Ni and cobalt ion (Co) transporters, involved in the absorption process of Ni by cells [10]. The *AtIRT1* (iron-regulated transporter 1) gene, as an important metal ion absorption transporter, when knocked out, leads to a deficiency of Ni and is accompanied by cell differentiation defects, indicating that this gene directly regulates the absorption and transport processes of Ni [11]. The NRAMP (natural resistance-associated macrophage protein) family genes are responsible for the transport of various metal ions within plants, playing a crucial role in maintaining the homeostasis of Ni in plants [12]. The ZIP (Zn-regulated, iron-regulated transporter-like protein) family genes have transmembrane structures and characteristics, typically promoting the transport of cations into the cytoplasm to facilitate metal ions (ferrous ion/Fe^2+^, zinc ion/Zn^2+^, manganous ion/Mn^2+^, and nickel ion/Ni^2+^) steady state, and it was found that under the condition of Fe deficiency, the content of Ni was significantly reduced and the cell differentiation ability was significantly weakened, indicating that this family of genes had a biological process of the simultaneous regulation of Fe and Ni transport [13,14].

Wheat, as an important staple crop for humans, provides about 20% of the energy and protein in human diets (data from WHO). Once contaminated with heavy metals such as Ni, it can pose a serious threat to human health [15]. Therefore, developing wheat varieties that can withstand Ni stress and maintain an appropriate balance of Ni levels through biofortification is crucial for improving wheat yield and quality. Like other trace element traits, the absorption and transport of Ni are typical quantitative traits controlled by multiple genes, showing continuous phenotypic variation in natural populations [15,16,17]. However, to date, only two studies have reported on the genetic locus mapping of Ni content in wheat grains, providing very limited data on the identification of major loci for Ni accumulation in wheat grains and the screening of superior allele genotypes. Specifically, Bhatta et al. used a synthetic hexaploid wheat population (*Triticum durum* L. × *Aegilops tauschii Coss*) consisting of 123 lines and 35,939 valid single nucleotide polymorphism (SNP) markers to identify eight SNP loci significantly associated with wheat grain Ni content on chromosomes 1A, 2D, 3A, 4D, 5B, and 6A [17]. In contrast, Safdar et al. used a natural population of 120 common wheat samples and 90K array genotyping data to identify one such locus on chromosome 1A. The hotspot region of QTN was significantly associated with grain Ni content, and four SNPs with low effect values were identified on chromosomes 3B and 4B, which were also significantly associated with grain Ni content [18].

Despite previous efforts to identify genetic loci controlling Ni accumulation in wheat grains using different populations, the genetic and molecular mechanisms of Ni accumulation in wheat grains remain unclear. Using naturally occurring populations with rich genetic diversity and higher-resolution genotyping techniques can help identify the quantitative trait loci that have significant genetic effects on Ni accumulation in wheat grains. This study utilized natural populations comprising 207 wheat varieties and Axiom^®^ Wheat660 Genotyping Arrays genotyping data to identify quantitative trait loci controlling Ni accumulation in wheat grains using GWAS methods. The main objectives of this study are (1) to elucidate the genetic mechanisms controlling Ni accumulation in wheat grains; (2) to identify candidate genes that may be involved in regulating Ni accumulation in wheat grains; (3) to evaluate the genetic effects of major effect loci, identify superior haplotypes, and guide research on biofortification breeding for Ni in wheat grains.

## 2. Results

### 2.1. Phenotypic Variation in Ni Content in Wheat Grains in Natural Population

**Figure 1:**
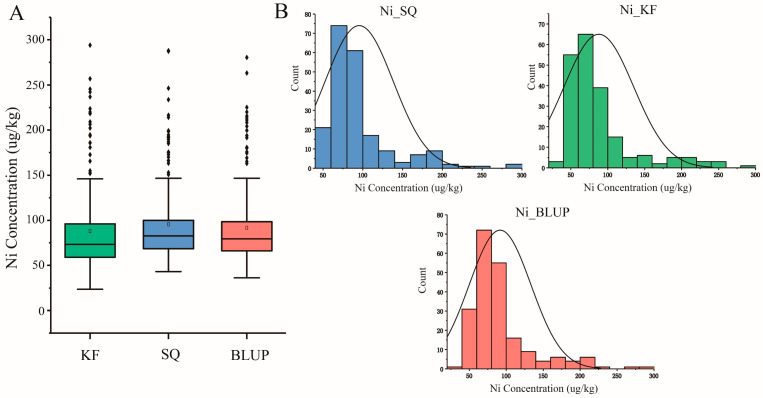
Distribution of Ni content in the panel of association population under different environments: (**A**) Boxplot of Ni grain content in SQ, KF, plus the BLUP of all the environments; (**B**) distribution of the Ni content in the association populations from KF, SQ, and BLUP.

A survey was conducted on the Ni content in grains of natural population materials planted in SQ (Shangqiu) and KF (Kaifeng) in 2022. It was found that there is a significant variation in the Ni content of wheat grains at the population level in each environment (Figure 1A). In the natural population planted in SQ, the variation range of the Ni content in wheat grains is 43.13–287.93 μg/kg, with an average grain Ni content of 95.42 μg/kg. In the natural population planted in KF, the variation range of the Ni content in wheat grains is 23.46–294.05 μg/kg, with an average grain Ni content of 87.85 μg/kg. The best linear unbiased prediction value (BLUP) derived from phenotypic data in both environments has a variation range of 36.28–280.47 μg/kg (Table 1).

From the distribution of phenotypic values at the population level, the phenotypic value distribution in each environment (including BLUP) follows a normal distribution, indicating that this population is ideal for analyzing the association of the Ni content in wheat grains (Figure 1B). Additionally, using the Pearson correlation coefficient method, a correlation analysis was performed on the phenotypic values of natural populations planted in SQ and KF, with a correlation coefficient of 0.91 (Figure S1), and the broad genetic ability is H^2^ = 0.944, indicating that the Ni content in wheat grains is mainly influenced by genetic effect.

### 2.2. GWAS of Ni Content in Wheat Grains

Before performing the GWAS, the 660K SNP genotyping assay was filtered in the PLINK software. After frequency and genotyping pruning [the minor allele frequency (MAF) is greater than 0.05 and the genotype missing data are less than 10%], there were 244,508 SNPs. Finally, 207 cultivars with 244,508 SNPs were retained for further analysis. The results of the principal component analysis (PCA) indicate that there is no significant population stratification within the association panel. The first five principal components, which account for 70.23% of the genetic variation among the samples, can be used as covariates for the subsequent GWAS (Figure S2, Table S1).

To enhance the reliability of the marker–trait association (MTA) analysis, three models were employed to conduct the GWAS for the Ni concentration in wheat grains across two locations and the BLUP. These three models include the general linear model (GLM), the mixed linear model (MLM), and the fixed and random model circulating probability unification (FarmCPU). A total of 75 SNPs were identified in all the environments to be significantly associated with the Ni content in wheat grains (threshold standard *p*-value < 1.0 × 10^−4^). Among them, 29 SNPs were commonly identified in the three analysis models (Figure S3). According to the physical positions of the SNP markers, they could be combined into five genetic loci controlling the Ni content in wheat grains (Table 2).

These loci were mainly distributed on chromosomes 3A, 4B (2 loci), and 6B (2 loci), and could explain 8.20–11.29% of the phenotypic variation (PVE). Among them, three loci involving three peak SNPs were detected in at least two environments, and these loci were stable genetic loci under multiple environmental conditions. In addition, SNPs AX-111126872 and AX-110161827 were identified by the GLM, MLM, and FarmCPU models in all the environments. Among them, the peak SNP AX-111126872, which was located on chromosome 6B, exhibited the highest PVE in each environment, ranging from 9.43 to 11.29%. Furthermore, a QTL hotspot exists at the beginning of chromosome 6B. This locus could be a key factor for wheat grain Ni accumulation.

**Figure 2:**
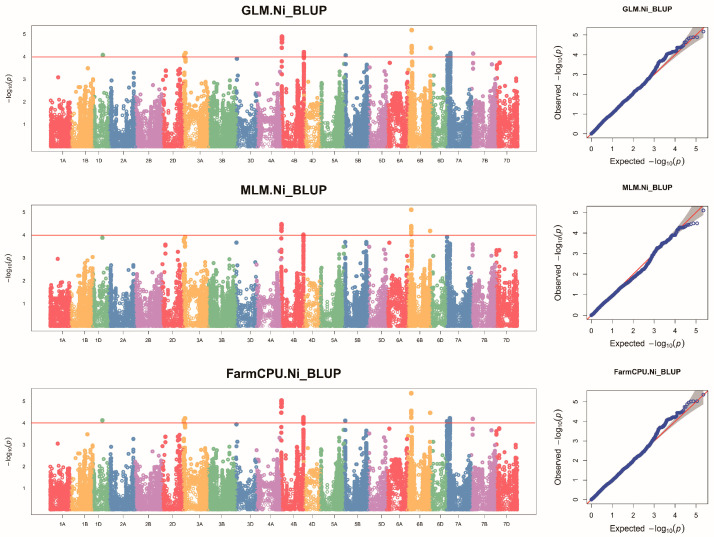
Manhattan and QQ plots for Ni concentrations in wheat grains were generated using the GLM, MLM, and FarmCPU models within the BLUP framework. The dashed horizontal line indicates the significance threshold of −log_10_(P) = 4.0. SNPs above the red dotted line are significantly associated with Ni variation.

After comparing with previous research results, a genetic locus was found to have co-localization, specifically a QTN *AX-110161827* on chromosome 4B that co-localizes with *qNi14*. However, two additional genetic loci identified in this study on chromosome 6B, which are stably present under multiple environments and have higher effect values, have not been reported before. These are newly identified genetic loci controlling Ni accumulation in wheat grains, and the comparison results are listed in Table 3. The Manhattan plot and quantile–quantile (QQ) plot of the GLM, MLM, and FarmCPU statistical models of wheat grain Ni accumulation in the BLUP environment are illustrated in Figure 2. The Manhattan plots and QQ plots of the remaining environments for the GLM, MLM, and FarmCPU models are shown in Figures S4–S6, respectively. The information regarding significant SNPs of the GLM, MLM, and FarmCPU models is presented in Tables S2–S4, respectively.

### 2.3. Prediction of the Candidate Genes for Genetic Loci Controlling Ni Accumulation

In this study, using different GWAS models, three peak SNPs were identified to be significantly associated with Ni accumulation in wheat grains under multiple environmental conditions and remain stable. Using the wheat genome reference sequence (IWGSC Ref-Seq v1.1), public database of wheat expression profiles [http://www.wheat-expression.com (accessed on 25 November 2024)], and grain expression data from 207 populations after 20 days after pollination (Data Availability Statement), the candidate genes related to Ni accumulation in wheat grains at these three stable genetic loci are worth noting (Table 3 and Table S5). The SNP AX-111126872 (6B) has the highest association with Ni accumulation in wheat grains (PVE, 9.43–11.29%), with a *TraesCS6B02G132400* gene located on its side. This gene is expressed at the highest level in grains, approximately six times that of other organs, encoding *GRPs*, and plays crucial roles in the response to heavy metal stress. Additionally, another SNP AX-108754008 significantly associated with Ni accumulation in wheat grains is near a gene *TraesCS6B02G423100* encoding *glutathione reductase* (*GR*), which regulates glutathione biosynthesis and plays a role in Ni tolerance in several plant species. Compared to other genes, this gene has a higher expression level in wheat grains. Near the 4B chromosome AX-110161827 locus, there are two genes encoding transmembrane proteins, *TraesCS4B02G014100* and *TraesCS4B02G019000*, both of which encode *membrin protein* and *transmembrane protein 56* (*TMP56*), respectively. Only *TraesCS4B02G019000* shows a significant difference in expression levels compared to superior and inferior alleles (*p*-value = 4.17 × 10^−7^), predicting it as a candidate gene for the AX-110161827 locus (Figure S7, Table S6).

**Figure 3:**
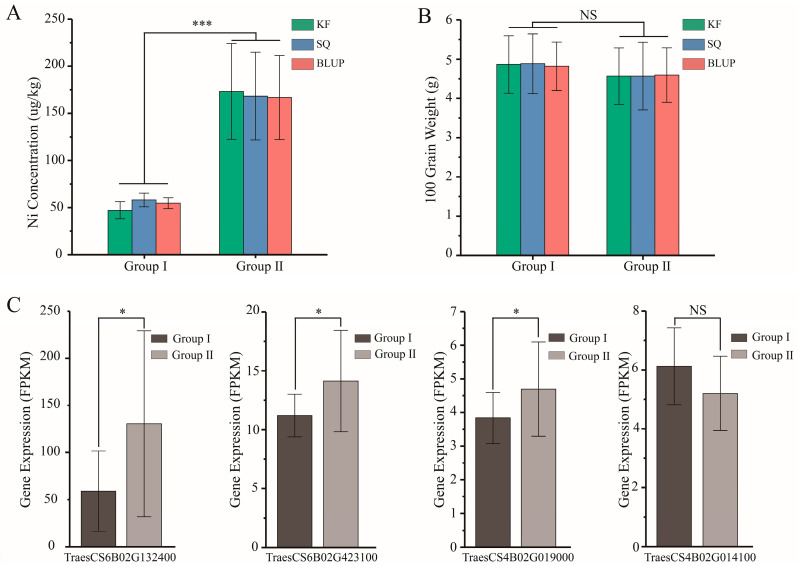
Analysis of the global gene expression in high- and low-Ni individual lines: (**A**) Ni concentrations in wheat grains of high-Ni and low-Ni groups; Group I contains 15 individual lines with high Ni concentration, and Group II contains 15 individual lines with low Ni concentration. (**B**) There is no significant difference in the 100-grain weight between Group I and Group II in different environments. (**C**) Analysis of the differences in the expression levels of candidate genes between Group I and Group II. * and *** indicate that the expression levels of the candidate genes are significantly different between the two groups (* indicates *p*-value < 0.05 and *** indicates *p*-value < 0.001), and NS indicates that there is no difference in the expression levels of the candidate genes between the two groups.

To further identify the potential genes that may regulate Ni accumulation in wheat grains, we analyzed the expression patterns of the candidate genes. To avoid the influence of biomass on the Ni content in wheat grains, we selected individual lines with no significant difference in 100-grain weight but extreme differences in grain Ni content (15 individual lines per group) (Table S7). Using expression profile data from 20 days after grain filling (Data Availability Statement), we analyzed the expression levels of the candidate genes. The results showed that the average Ni content in the grains of high-Ni individual lines (Group II) in the KF, SQ, and BLUP environments were 173.34 ± 50.76, 168.36 ± 46.56, and 166.80 ± 44.55, respectively, while the average 100-grain weight were 4.57 ± 0.72, 4.57 ± 0.86, and 4.59 ± 0.70, respectively. In contrast, the average Ni content in grains of low-Ni individual lines (Group I) in KF, SQ, and BLUP environments were 47.17 ± 9.05, 58.08 ± 7.35, and 54.62 ± 5.78, respectively, with an average 100-grain weight of 4.87 ± 0.73, 4.88 ± 0.76, and 4.82 ± 0.62, respectively (Table S8). The results of the difference analysis indicated that there was no significant difference in the 100-grain weight between Group I and Group II, while the Ni content showed an extremely significant difference (Table S8). Subsequently, the expression levels of candidate genes in Group I and Group II were analyzed. It was found that the expression levels of *TraesCS6B02G132400*, *TraesCS6B02G423100*, and *TraesCS4B02G019000* were significantly lower in Group I compared to Group II, while there was no significant difference in the expression level of the *TraesCS4B02G014100* between Group I and Group II (Figure 3C, Table S9). Therefore, it is inferred that *TraesCS6B02G132400*, *TraesCS6B02G423100*, and *TraesCS4B02G019000* may be involved in the accumulation of Ni in wheat grains.

### 2.4. Phenotypic Effect Evaluation of the Three Stable Genetic Loci

**Figure 4:**
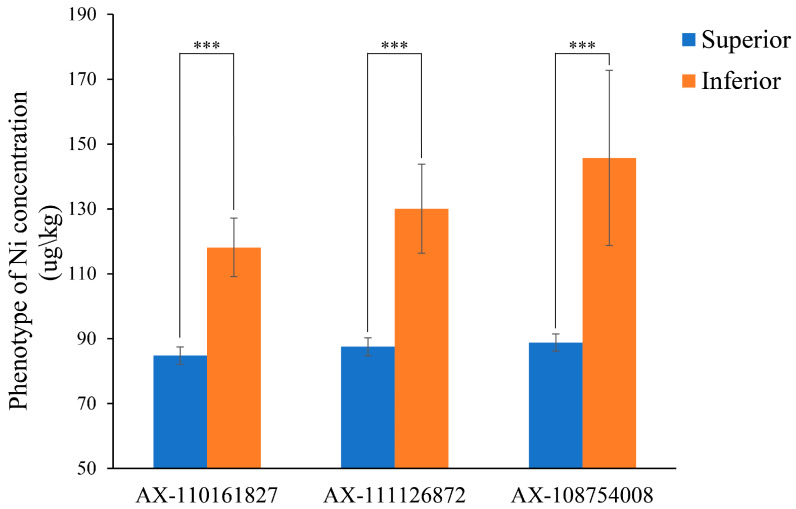
The phenotypic performance of cultivars with superior and inferior alleles at three repetitive significantly associated SNP loci for grain Ni content. The blue bars denote the phenotype values of superior alleles, while the brown bars represent the phenotype values of inferior alleles. The figure was generated using BLUP values, with *** indicating a *p*-value < 0.0001 and the error bars in the figure representing the standard error (SE).

All three peak SNPs identified by GWAS with different statistical models in at least two environments were considered the stable loci for controlling Ni accumulations in wheat grains. This study aimed to evaluate the genotypes and corresponding phenotypes of the three stable SNPs in natural populations using an analysis of variance (ANOVA). For each of these three SNPs, varieties carrying superior alleles had lower Ni concentrations in their grains compared to those carrying inferior alleles (Figure 4). Statistical analysis showed that under all the environmental conditions, the difference in the Ni content in grains between materials carrying superior alleles and those carrying inferior alleles reached extremely significant levels (Figure 4, Table 4).

Specifically, the QTN AX-110161827 is located on chromosome 4B, the superior allele is TT, and the inferior allele is CC. The difference in phenotypic values reaches a highly significant level across all the environments (*p*-value < 0.01). Notably, this study identified two new QTNs, AX-111126872 and AX-108754008, located on chromosome 6B. The differences between the superior and inferior alleles of these two QTNs (the superior alleles of AX-111126872 and AX-108754008 are CC and AA, respectively, and inferior alleles are TT and GG, respectively) were more substantial, being 42.52 and 56.9, respectively (the BLUP differences among different haplotypes) (Table 4). Both differences reached a highly significant level, indicating that these novel loci have a stronger effect and thus greater value for breeding applications.

## 3. Discussion

Ni is an essential micronutrient for plants, playing crucial roles in various metabolic processes. It is needed in very small amounts compared to macronutrients [19,20]. To date, several genes related to the accumulation of Ni in plants have been identified in various plants [21,22]. However, the accumulation of Ni in wheat grains is still poorly understood. Thus, we carried out GWAS to dissect the genetic architecture of Ni accumulation in wheat grains, providing insights into the underlying mechanisms and potential targets for breeding wheat varieties without Ni toxicity.

### 3.1. High-Density Molecular Markers and Genetic Diversity

The high-density molecular markers and the high genetic diversity play crucial roles in identifying the genetic loci through genome-wide association study methods [23,24]. Previous researchers have developed several wheat assays for genotyping at the whole-genome level, including assays with 35K, 90K, 660K, and 820K [24,25,26]. However, compared to other chips, the SNP distribution of the 660K assay is more extensive, annotating almost all the genes that other assays annotate, exhibiting reliable and cost-effective characteristics [24]. Therefore, this chip is considered an ideal choice for genotyping in population materials. In this study, we used a wheat 660K SNP assay to genotype population materials, filtering down to 244,508 SNPs used for the GWAS, which is a substantial number compared to previous studies. For instance, Bhatta et al. used 35,939 SNPs to identify genetic loci associated with Ni accumulation in wheat, while Safdar et al. used 90K assay data [17,18]. Additionally, the number of populations is a critical factor constraining GWAS; more populations may mean greater genetic diversity at the population level, which is beneficial for identifying the genetic loci that control complex trait expression [27,28,29]. In this study, natural populations composed of 207 materials were used for genotyping. We determined the genetic locus controlling Ni accumulation in wheat grains. Although the population size is not sufficiently large [30,31,32] and is relatively small compared with other studies, the current population of wheat grains contains dramatic phenotypic variations, ranging from 23.46 μg/kg to 294.05 μg/kg, and it shows a normal distribution of Ni content, suggesting that the population has rich genetic diversity, which is conducive to GWAS.

### 3.2. Determining High-Confidence Genetic Loci Through Multi-Model GWAS

Previous research has shown that the content of trace elements in crop seeds is influenced by both the environment and genes [17,33,34,35]. The magnitude of broad-sense heritability has significant impacts on the explanatory power of GWAS, the reliability of results, the choice of research strategies, and the understanding of missing heritability [36,37]. For example, Madhav et al. found that the broad-sense heritability of copper, iron, magnesium, manganese, and zinc content in wheat grains was all greater than 0.650, and they identified a number of genetic loci regulating the accumulation of trace elements using GWAS [17]. In this study, the broad-sense heritability of the Ni content in wheat grains was 0.944, indicating that genetic factors play a dominant role in determining Ni accumulation in wheat grains. Moreover, to enhance the reliability of the GWAS results, this study employed three analysis models (GLM, MLM, and FarmCPU) to analyze the Ni content in wheat grains. A total of 29 SNPs were identified by all three models, which can be merged into five loci. Among them, AX-110161827 on chromosome 4B (11.59 Mb) and AX-111126872 and AX-108754008 on chromosome 6B (125.48 Mb and 693.58 Mb, respectively) were identified across multiple environments, indicating stable genetic loci. Although there are limited reports on Ni accumulation in wheat grains, the QTN AX-110161827 identified in this study is co-localized with the *qNi14* on chromosome 4B reported by Safdar et al., implying good repeatability of QTLs controlling Ni accumulation in wheat grains [18]. It is worth noting that a QTL hotspot was identified at the front end of chromosome 6B in this study. The peak SNP AX-111126872, with the highest PVE (ranging from 9.43% to 11.29% across all the environments), could not be associated with any previously reported QTL for Ni grain content. This indicates that by using the population selected in this study, we were able to identify a novel stable QTL associated with Ni accumulation in wheat grains, which may harbor a major gene regulating Ni accumulation in wheat grains.

### 3.3. Putative Candidate Genes for Ni Accumulation

In this study, GWAS employing various statistical models across multiple environments identified three peak SNPs and their corresponding loci associated with Ni accumulation (Tables S2–S4). By integrating physical position data, functional annotations, and gene expression patterns (Figure S7, Table S5), we pinpointed three genes as the most likely candidates influencing Ni accumulation (Table 3, Tables S5 and S6). Initially, a hotspot locus associated with 11 significant SNPs was identified at the beginning of chromosome 6B. The peak SNP AX-111126872, which exhibited the highest average PVE for Ni concentration across all the environments, points to *TraesCS6B02G132400*—a gene encoding *GRPs*—as a critical factor influencing Ni accumulation in wheat grains. Previous studies have demonstrated that the expression of *AtGRP7* is significantly upregulated by Ni stress in Arabidopsis. This upregulation is associated with enhanced Ni tolerance in plants [38]. Although there is no direct evidence that *GRP7* interacts with a specific Ni transporter in the context of seed Ni content, it is known that *GRP7* may directly or indirectly modulate the expression of the genes associated with heavy metal chelators, such as metallothioneins (MTs) and phytochelatins (PCs), which sequester excess Ni to mitigate its toxicity [39]. Furthermore, the elevated Ni accumulation in seeds may arise from a systemic physiological process involving long-distance transport and sequestration from other tissues. Given *GRP7*’s role in RNA metabolism and stability, it can post-transcriptionally regulate genes governing Ni translocation and storage. Additionally, *GRP7* may modulate the activity of key antioxidant enzymes—such as superoxide dismutase (SOD), catalase (CAT), and peroxidase (POD)—thereby enhancing the plant’s tolerance to Ni-induced oxidative stress [38,40]. The above-mentioned clues imply that *GRP7* likely modulates Ni uptake and partitioning in plants through dual mechanisms involving transporter regulation and post-transcriptional control. In this study, *TraesCS6B02G132400*, a putative *GRP7* homolog, exhibited the highest expression level in wheat grains, surpassing its expression in other tissues by more than sixfold (Table S5). Moreover, in high-Ni individual lines (Group II), the expression level of the *TraesCS6B02G132400* gene in the grains is much higher than that in low-Ni individual lines (Group I), nearly three times as high, and the difference reaches a significant level (Figure 3C, Table S9). This may be attributed to the fact that *TraesCS6B02G132400* promotes Ni accumulation in seeds by regulating the expression of antioxidant enzyme genes and heavy metal chelator genes. This accumulation could be a wheat adaptation strategy to sequester Ni in specific tissues (e.g., grains) rather than in flag leaves, stems, or other organs, thereby reducing Ni’s potential toxicity to critical physiological processes. Another key SNP AX-108754008 is located on chromosome 6B and corresponds to a gene *TraesCS6B02G423100* encoding *GR*. This enzyme is implicated in Ni accumulation mechanisms. It has been demonstrated by previous studies that Ni is transported through plants via several mechanisms, primarily involving chelation and sequestration. In hyperaccumulator plants, such as those in the genus Thlaspi, Ni is often chelated by amino acids like histidine and nicotianamine, which facilitate its transport from roots to shoots [41]. Once Ni reaches the aerial parts of the plant, it can be sequestered into vacuoles to prevent toxicity. This sequestration is mediated by vacuolar metal-ion transporter proteins. For instance, the increased expression of metal tolerance proteins (MTPs) has been associated with Ni sequestration in vacuoles, thereby reducing its toxicity in the cytoplasm [42]. Additionally, the specific chelator used can vary among hyperaccumulator species. The citrate is the primary ligand for Ni in some New Caledonian hyperaccumulators, while malate is more prevalent in Brassicaceae species [43,44]. In addition to chelation and sequestration, glutathione (GSH) plays a significant role in Ni accumulation. In Thlaspi goesingense, high concentrations of GSH coincide with constitutively high activity of serine acetyltransferase (SAT) and *GR* [45]. This elevated GSH biosynthesis helps protect against Ni-induced oxidative damage and enhances Ni tolerance. In addition, a positive correlation between GSH levels and Ni accumulation has been observed in various Thlaspi species [46]. Given these findings, the transcriptional level of *GR* may significantly influence the accumulation of Ni in wheat grains. In our study, we identified a key SNP (AX-108754008) on chromosome 6B in wheat, which corresponds to a gene encoding *GR* (*TraesCS6B02G423100*), and that the *TraesCS6B02G423100* gene exhibited relatively high expression levels in both wheat grains and other tissues (Table S5). Meanwhile, in high-Ni individual lines (Group II), the expression level of the *TraesCS6B02G423100* was significantly higher than that in low-Ni individual lines (Group I), suggesting that the expression level of this gene may be positively correlated with the accumulation of Ni in wheat grains (Figure 3C, Table S9). The remaining SNP AX-110161827 on chromosome 4B was associated with two candidate genes *TraesCS4B02G014100* and *TraesCS4B02G019000*, both encoding membrane proteins of *membrin protein* and *TMP56*, respectively. However, the results of a haplotype analysis showed that only *TraesCS4B02G019000* exhibited a significant difference in expression between the superior haplotype cultivars and the inferior haplotype cultivars (Table S6). At the same time, in high-Ni individual lines (Group II), only the expression level of the *TraesCS4B02G019000* gene was significantly higher than that in low-Ni individual lines (Group I), while there was no significant difference in the expression level of the *TraesCS4B02G014100* gene between the Group I and the Group II (Figure 3C, Table S9). Therefore, it is inferred that *TraesCS4B02G019000* is a reliable candidate gene for the QTN AX-110161827 on chromosome 4B. Although specific studies on *TMP56* are limited, transmembrane proteins participate in various physiological activities in plants, including substance transport and stress responses [47,48,49]. Additionally, other well-studied membrane proteins like ABC transporters (ATP-binding cassette transporters) have been shown to be involved in the transport of heavy metal ions highlighting the importance of membrane proteins in ion homeostasis and stress tolerance [50]. Therefore, *TMP56* likely contributes to ion transport and stress response in plants through similar mechanisms. While GWAS has provided valuable insights into the genetic basis of Ni accumulation in wheat, several challenges remain. The complex nature of Ni homeostasis, involving multiple genes and pathways, requires further functional validation of candidate genes. Additionally, future research should also focus on the development of functional markers and the application of gene editing technologies to precisely modify genes involved in Ni metabolism.

### 3.4. Molecular Pyramiding Breeding for Ni Accumulation in Wheat

Ni accumulation in wheat grain poses significant public health risks due to its potential toxicity upon dietary exposure. Chronic intake of Ni-contaminated wheat has been linked to allergic dermatitis, respiratory disorders, and nephrotoxicity in humans, even at low concentrations [51]. Wheat, as a global staple, contributes substantially to dietary Ni intake, particularly in regions with contaminated soils or industrial pollution [52]. The European Food Safety Authority (EFSA) established a Ni tolerable daily intake (TDI) of 2.8 μg/kg body weight, emphasizing the need to monitor grain Ni levels (EFSA, 2015). Previous findings demonstrate that Ni translocation to grains is influenced by soil pH and cultivar-specific uptake efficiency [53,54]. When the Ni content in wheat grains is high, it can exceed the safety threshold and pose a threat to human health. Therefore, breeding efforts should prioritize restricting Ni translocation to edible parts while maintaining yield, as even subtoxic Ni levels may synergize with other dietary metals to exacerbate health risks [55]. Furthermore, excessive Ni accumulation disrupts wheat physiology, leading to reduced growth, chlorosis, and impaired nutrient uptake (e.g., iron and magnesium) due to oxidative stress and enzyme inhibition [56]. At high concentrations, Ni toxicity decreases photosynthetic efficiency by damaging chloroplasts and Rubisco activity, directly reducing biomass and tiller formation [56,57]. Yield losses arise from diminished grain number and size, as Ni interferes with reproductive development, including pollen viability and grain filling [58]. Additionally, Ni excess alters grain quality by altering protein and starch composition, as well as increasing Ni translocation to edible parts, raising concerns for both nutritional and food safety standards [52]. These impacts highlight the need for breeding wheat with restricted Ni uptake or enhanced detoxification traits. In this study, our GWAS identified three stable QTNs on chromosomes 3A (AX-110161827), 4B (AX-111126872), and 6B (AX-108754008) associated with reduced Ni accumulation in wheat grains. These loci exhibited consistent effects across environments, with lines carrying superior alleles (TT, CC, and AA, respectively) showing significantly lower grain Ni concentrations (*p*-value < 0.01) compared to those with inferior alleles (CC, TT, and GG, respectively). The significant SNPs can be converted into Kompetitive Allele-Specific PCR (KASP) markers for molecular breeding [59]. These KASP markers can be directly applied in wheat breeding programs. By screening germplasm collections or breeding populations, breeders can efficiently select lines carrying the superior alleles for Ni accumulation. Then, breeders should introgress individual QTNs into lines with excellent agronomic performance [60], and combine them through successive crosses while monitoring Ni accumulation and agronomic performance in the progeny. The ultimate goal is to develop wheat varieties that not only have reduced Ni accumulation but also maintain high yield and elite grain quality. By integrating marker-assisted selection (MAS) with traditional breeding, breeders can accelerate the development of such varieties, thereby addressing both agronomic and food safety concerns.

## 4. Materials and Methods

### 4.1. Plant Materials

The natural population materials used in this study consist of 207 hexaploid wheat (*Triticum aestivm* L.) varieties, sourced from different wheat-growing regions in China (the North Huaihai region, the South Huaihai region, and the middle and western Yangtze River region), as well as seven other countries. A variety of materials were obtained through a joint laboratory project between the Henan Academy of Agricultural Sciences (HAAS, Zhengzhou, Henan, China) and the International Maize and Wheat Improvement Center (CIMMYT, Mexico City, Mexico). The materials are derived from the Henan Crop Germplasm Bank (HAAS, Zhengzhou, Henan, China), which is constructed and managed by the Henan Academy of Agricultural Sciences, and the use of these materials has been approved with necessary permits. The natural population materials were collected in KF City, Henan Province, from 2022 to 2023 (KF, 114°30′ E, 34°80′ N) and SQ City (SQ, 115°65′ E, 34°45′ N), and a randomized block field trial design was used for planting. Each variety was planted in a plot measuring 2 m long and 0.2 m wide, with a spacing of 10 cm between plants. A total of 42 plants were grown in two rows in each plot. Three replications were maintained for each field plot under all the environmental conditions. Sowing took place in October, and harvesting occurred in June of the following year. All the experimental materials were planted according to standard field management practices.

### 4.2. Determination of Ni Content and 100-Grain Weight in Wheat Grains

The mature wheat grains were harvested in small plots, threshed, and dried at 55 °C for 24 h. They were then ground into a fine powder. After grinding, the powdered samples were further dried at 55 °C for 24 h. Subsequently, 200 mg of the dried powder from each sample was accurately weighed and placed in 8.0 mL of nitric acid, which was then dissolved in a microwave reactor for (AIM500 Digestion System, A.I. Scientific, Canberra, Australia) digestion. The (AIM500 Digestion System, A.I. Scientific, Canberra, Australia) digestion process used a temperature gradient from 120 °C to 180 °C, lasting 30 min. After dilution in deionized distilled water, the Ni concentration was measured by inductively coupled plasma mass spectrometry (ICP-MS, NexION 1000, Perkin Elmer, Waltham, MA, USA). Additionally, in the two environments of KF and SQ, 10 uniformly sized spikes were carefully selected from each individual line within the natural population. Subsequently, 100 seeds were randomly picked from the grains of these selected spikes, and their weight was measured. The weighing process was carried out continuously three times, and the average value was taken as the final 100-grain weight data.

### 4.3. Genotyping and Quality Control

High-quality genomic DNA was isolated by CTAB from the leaves of young seedlings belonging to the 207 wheat varieties within the association population. Then, using Axiom^®^ Wheat660 Genotyping Arrays [http://www.cgmb.com.cn/index.php/Home/Index/ke_suc_case_xiangqing?id=22#:~:text=Axiom%C2%AE%20Wheat660Genotyping (accessed on 19 May 2024)], according to the protocol specified in Axiom 2.0 Assay Manual Work-flow protocol, the DNA was genotyped. Following the recommendations in the Plink 2.0 software [https://www.cog-genomics.org/plink/2.0/ (accessed on 15 March 2024)], the following settings were used to filter the raw data: “--maf 0.05--geno 0.1”, which means that MAF is greater than 0.05 and the genotype missing data are less than 10% [24,51]. A total of 244,508 SNP markers were used for genome-wide association studies and subsequent analyses.

### 4.4. Statistical Analysis and GWAS Mapping

Using R software version 4.2.2 [https://cran.r-project.org/bin/windows/base/old/4.4.2/ (accessed on 1 November 2024)], the frequency distribution of phenotypic values in the population was plotted. In the R environment, with the help of the “psych” package, descriptive statistical analysis was conducted on all the phenotypic data from various planting sites, covering parameters such as mean, range, and normal distribution descriptors like skewness and kurtosis. By fitting a mixed linear model using the R package “lem4”, the BLUP values for each variety at two planting sites were obtained, and calculated according to the following formula: Y = (1|Line) + (1|Loc) + (1|Rep% in% Line: Loc) + (1|Line: Loc). Ultimately, the BLUP values of Ni content and 100-grain weight for each material in both planting environments were used as input data for subsequent linkage analysis. The Pearson correlation coefficient (r) for Ni content in wheat grains from two environments was also calculated using R 4.2.2.

GWAS were implemented by GAPIT packages in the R software [61]. The analysis employed an MLM approach incorporating both principal components and kinship matrix (K) to account for population structure and genetic relatedness [62]. The variance—covariance K was calculated using the VanRaden method [63]. The first five principal components of the SNP data were included in the GWAS model (Figure S2, Table S1). In order to identify the most suitable statistical model, three models were utilized to explore the associations between the phenotypic and genotypic datasets. These comprised the GLM, which solely considers the population structure; the MLM, which takes into account both the population structure and relative kinship; and the FarmCPU, which accounts for fixed and random effects and enhances the calculation speed and precision. A suggested *p*-value of 1.00 × 10^−4^ was adopted to regulate the genetic false positive error rate for this particular population [24,51]. The R package “CMplot” was used to visualize the Manhattan and QQ plots.

### 4.5. Putative Candidate Gene Predictions

The genomic sequences, which had a physical distance of 5 Mb both upstream and downstream of the peak SNP within all three integrated stable loci, were utilized for candidate gene predictions. In accordance with the previously reported common wheat reference genome sequence “IWGSC RefSeq v1.1” of the variety Chinese Spring, a catalog of high-confidence genes was retrieved from the International Wheat Genome Sequence Consortium (IWGSC) at https://wheatgenome.org (accessed on 19 November 2024). This gene list was then utilized to seek out the potential candidate genes within each locus. The annotation of candidate genes was carried out with the aid of Ensemble Plants [http://plants.ensembl.org/Triticum_aestivum/Info/Index (accessed on 20 November 2024)]. The physical coordinates of each SNP originating from the 660K arrays were acquired from the IWGSC website [https://www.wheatgenome.org (accessed on 19 November 2024)]. Subsequently, the genes that exhibited expression in grains were sieved out from the Wheat Expression Browser [http://www.wheat-expression.com (accessed on 25 November 2024)], and the RNA-seq data are available in Genome Sequence Archive [https://bigd.big.ac.cn/gsa/browse/CRA004223 (accessed on 29 November 2024)]. These filtered genes were then employed to conduct a comparison of the expression levels between the alleles of each peak SNP within the association population. Only those genes that manifested expression in grains 20 days after pollination and demonstrated a significant differentiation (*t*-test, with a *p*-value less than 0.05) between the superior and inferior alleles were taken into account. Then, the candidate genes were predicted by following the functional annotation.

## 5. Conclusions

In conclusion, GWAS offers a robust framework for identifying beneficial genetic variants associated with Ni accumulation in wheat grains. By leveraging these insights, researchers and breeders can work towards developing wheat varieties that are not only nutritionally enhanced but also resilient to environmental challenges.

## Figures and Tables

**Table 1:** Descriptive statistics of the Ni contents in the association population.

Location	Year	Trait	Mean ± SD ^1^ (μg/kg)	Range (μg/kg)	Kurt ^2^	Skew ^3^
Kaifeng (KF)	2023	Ni content	95.42 ± 43.03	43.13–287.93	4.33	1.99
Shangqiu (SQ)	2023	Ni content	87.85 ± 47.18	23.46–294.05	3.78	1.98
BLUP	2023	Ni content	91.63 ± 41.78	36.28–280.47	4.18	2.02

^1^ SD, Standard deviation. ^2^ kurtosis, often referred to as kurt, is a statistical measure that describes the “tailedness” of the probability distribution of a real-valued random variable. ^3^ skewness, also known as skew, is a measure of the asymmetry of the probability distribution of a real-valued random variable around its mean.

**Table 2:** A list of significant loci and their detailed information for Ni content identified by GWAS.

ID ^1^	Chr.	Interval Range (Mb)	No. of SNPs	Loc.	Peak SNP ^2^	Position (bp) ^3^	*p*-Value ^4^	R^2^ (%) ^5^
1	3A	15.42–50.74	9	SQ	AX-110424807	15,485,206	4.34 × 10^−5^	8.31
2	4B	6.64–11.66	7	KF	AX-110161827	11,593,889	4.84 × 10^−5^	9.76
				SQ	AX-110161827	11,593,889	3.18 × 10^−5^	8.63
				BLUP	AX-110161827	11,593,889	3.40 × 10^−5^	9.27
3	4B	670.43	1	BLUP	AX-109482775	670,432,861	9.70 × 10^−5^	8.20
4	6B	125.48–126.30	11	KF	AX-111126872	125,482,475	1.07 × 10^−5^	11.29
				SQ	AX-111126872	125,482,475	1.47 × 10^−5^	9.43
				BLUP	AX-111126872	125,482,475	7.94 × 10^−6^	10.77
5	6B	693.58	1	SQ	AX-108754008	693,584,519	4.94 × 10^−6^	10.58
				BLUP	AX-108754008	693,584,519	6.69 × 10^−5^	8.58

^1^ the ID of the loci identified through GWAS. ^2^ the most significant SNP on each locus. ^3^ the physical positions of the peak SNPs based on the IWGSC RefSeq v1.1 reference genome. ^4^ the *p*-values were calculated by the MLM. ^5^ the phenotypic variance explained by the MTA from the results of the MLM.

**Table 3:** Information for stable genetic loci associated with Ni accumulation identified via GWAS in current and previous studies.

ID ^1^	Chr.	SNP_Id	Position (Mb) ^2^	Region (Mb)	Near Locus Previously Reported in the Same Locus ^3^	Candidate Genes	Annotation
1	4B	AX-110161827	11.59	6.64–11.66	*qNi14* [18]	*TraesCS4B02G019000*	*Transmembrane protein 56*
2	6B	AX-111126872	125.48	125.48–126.30	--	*TraesCS6B02G132400*	*Glycine rich protein*
3	6B	AX-108754008	693.58	693.58	--	*TraesCS6B02G423100*	*Glutathione reductase*

^1^ the repetitive significantly SNP loci. ^2^ the physical positions of the SNP based on the IWGSC RefSeq v1.1 reference genome of Chinese Spring. ^3^ “--” indicated that there are no co-localized QTLs at this locus.

**Table 4:** ANOVA for individuals harboring the superior and inferior alleles for the stable significant SNPs in multi-environments.

SNP_Id	Chromosome	Allele Type		Phenotype Value (BLUP)		Allele Number		Allele Percentage (%)		*p*-Value		
		Superior	Inferior	Superior	Inferior	Superior	Inferior	Superior	Inferior	Ni_KF	Ni_SQ	Ni_BLUP
AX-110161827	4B	TT	CC	84.76	118.14	164	41	80.00	20.00	7.17 × 10^−6^	3.08 × 10^−6^	3.00 × 10^−6^
AX-111126872	6B	CC	TT	87.53	130.05	187	20	90.34	9.66	3.15 × 10^−5^	8.22 × 10^−6^	1.02 × 10^−5^
AX-108754008	6B	AA	GG	88.79	145.69	195	10	95.12	4.88	8.18 × 10^−4^	4.16 × 10^−7^	2.06 × 10^−5^

## Data Availability

The RNA-seq data of the wheat grains from the association population are available in the Genome Sequence Archive [https://bigd.big.ac.cn/gsa/browse/CRA004223 (accessed on 29 November 2024)].

## Supplementary Materials

The following supporting information can be downloaded at: https://www.mdpi.com/article/10.3390/plants14081262/s1, Figure S1: Correlation heatmap of phenotypic values of Ni content in wheat grains of the association population across different environments; Figure S2: Principal component analysis plots for the association panel comprising 207 wheat cultivars; Figure S3: The SNPs significantly associated with Ni content in wheat grains, which were repeatedly identified by the analysis models of GLM, MLM, and FarmCPU; Figure S4: Manhattan and QQ plots for Ni concentrations in wheat grains were generated using the GLM in all the environments. The dashed horizontal line indicates the significance threshold of −log10(P) = 4.0. SNPs above the red dotted line are significantly associated with Ni variation; Figure S5: Manhattan and QQ plots for Ni concentrations in wheat grains were generated using the MLM in all the environments. The dashed horizontal line indicates the significance threshold of −log10(P) = 4.0. SNPs above the red dotted line are significantly associated with Ni variation; Figure S6: Manhattan and QQ plots for Ni concentrations in wheat grains were generated using the FarmCPU model in all the environments. The dashed horizontal line indicates the significance threshold of −log10(P) = 4.0. SNPs above the red dotted line are significantly associated with Ni variation; Figure S7: Expression levels of promising candidate genes in different wheat tissues. The heat map was plotted using the transcripts per kilobase million (TPM) values from the gene expression profile public database; Table S1: The proportion of genetic variation explained by different principal components; Table S2: Marker-trait associations (MTAs) for Ni accumulation in the association population analyzed by the GLM; Table S3: Marker-trait associations (MTAs) for Ni accumulation in the association population analyzed by the MLM; Table S4: Marker-trait associations (MTAs) for Ni accumulation in the association population analyzed by the FarmCPU; Table S5: Differentially expressed genes annotation in all the integrated loci; Table S6: The expression of candidate genes among different haplotype groups associated with significant loci; Table S7: Raw data of the expression analysis of candidate genes in the groups with high and low Ni contents; Table S8: Analysis of the differences in 100-grain weight and Ni content between the groups with high and low Ni concentrations; Table S9: Analysis of the expression of candidate genes in the groups with high and low Ni contents; Table S10: The average phenotypic values for Ni accumulation across different environments in 207 wheat accessions, along with their BLUP estimates.

## Abbreviations

ANOVA	analysis of variance
BLUP	best linear unbiased predictors
CAT	catalase
CIMMYT	International Maize And Wheat Improvement Center
Co	cobalt
CTAB	Hexadecyl trimethyl ammonium bromide
DNA	Deoxyribonucleic Acid
EFSA	European Food Safety Authority
FarmCPU	fixed and random model circulating probability unification
Fe	ferrous
GLM	general linear model
GR	glutathione reductase
GRPs	glycine-rich proteins
GSH	glutathione
GWAS	genome-wide association studies
HAAS	Henan Academy Of Agricultural Sciences
IWGSC	International Wheat Genome Sequence Consortium
KASP	Kompetitive Allele-Specific PCR
KF	Kaifeng
MAF	minor allele frequency
MAS	marker-assisted selection
MLM	mixed linear model
Mn	manganous
MTA	marker–trait association
MTPs	metal tolerance proteins
MTs	metallothioneins
Ni	nickel
NRAMP	natural resistance-associated macrophage protein
PCA	principal component analysis
PCs	phytochelatins
POD	peroxidase
PVE	phenotypic variation explained
QQ	quantile–quantile
QTL	quantitative trait locus
QTN	Quantitative trait nucleotides
RNA	Ribonucleic acid
SAT	serine acetyltransferase
SNP	single nucleotide polymorphism
SOD	superoxide dismutase
SQ	Shangqiu
TMP56	transmembrane protein 56
ZIP	Zn-regulated, iron-regulated transporter-like protein
Zn	zinc
