# Supplementary material for: Unveiling Novel Genetic Loci and Superior Alleles for Nickel Accumulation in Wheat via Genome-Wide Association Study
Source: Plants (Basel). 2025 Apr 21;14(8):1262. doi: 10.3390/plants14081262 (PMC12030696; doi:10.3390/plants14081262)
Supplement: Supplementary file 1 [file plants-14-01262-s001.zip › plants-3519293-supplementary.pdf]

SUPPLEMENTAL INFORMATION

Figure S1. Correlation heatmap of phenotypic values of Ni content in wheat grains of the association population across different environments.

Figure S2. Principal component analysis plots for the association panel comprising 207 wheat cultivars.

Figure S3. The SNPs significantly associated with Ni content in wheat grains, which were repeatedly identified by the analysis models of GLM, MLM, and FarmCPU.

Figure S4. Manhattan and QQ plots for Ni concentrations in wheat grains were generated using the GLM in all the environments. The dashed horizontal line indicates the significance threshold of −log10(P) = 4.0. SNPs above the red dotted line are significantly associated with Ni variation.

Figure S5. Manhattan and QQ plots for Ni concentrations in wheat grains were generated using the MLM in all the environments. The dashed horizontal line indicates the significance threshold of −log10(P) = 4.0. SNPs above the red dotted line are significantly associated with Ni variation.

Figure S6. Manhattan and QQ plots for Ni concentrations in wheat grains were generated using the FarmCPU model in all the environments. The dashed horizontal line indicates the significance threshold of −log10(P) = 4.0. SNPs above the red dotted line are significantly associated with Ni variation.

Figure S7. Expression levels of promising candidate genes in different wheat tissues. The heat map was plotted using the transcripts per kilobase million (TPM) values from the gene expression profile public database.

Table S1. The proportion of genetic variation explained by different principal components

Table S2. Marker-trait associations (MTAs) for Ni accumulation in the association population analyzed by the GLM

Table S3. Marker-trait associations (MTAs) for Ni accumulation in the association population analyzed by the MLM

Table S4. Marker-trait associations (MTAs) for Ni accumulation in the association population analyzed by the FarmCPU

TableS5. Differentially expressed genes annotation in all the integrated loci

TableS6. The expression of candidate genes among different haplotype groups associated with significant loci

TableS7. Raw data of the expression analysis of candidate genes in the groups with high and low Ni contents

TableS8. Analysis of the differences in 100-grain weight and Ni content between the groups with high and low Ni concentrations

TableS9. Analysis of the expression of candidate genes in the groups with high and low Ni contents

Table S10. The average phenotypic values for Ni accumulation across different environments in 207 wheat accessions, along with their BLUP estimates

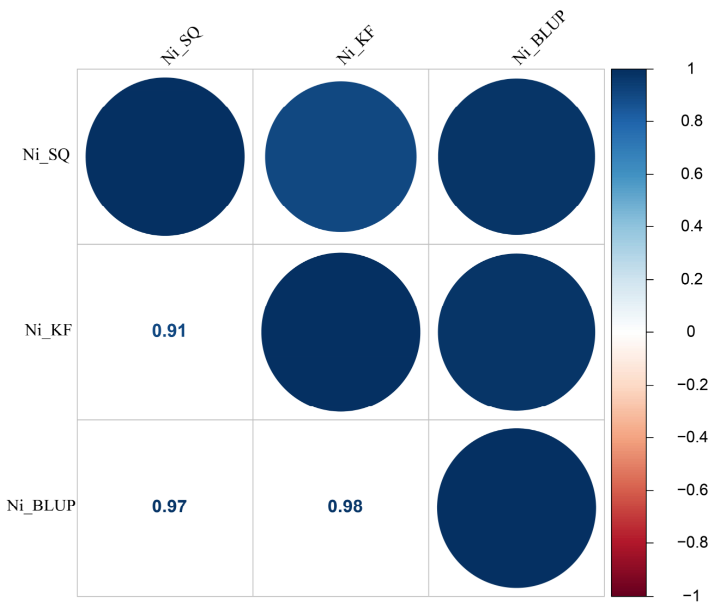

Figure S1. Correlation heatmap of phenotypic values of Ni content in wheat grains of the association population across different environments.

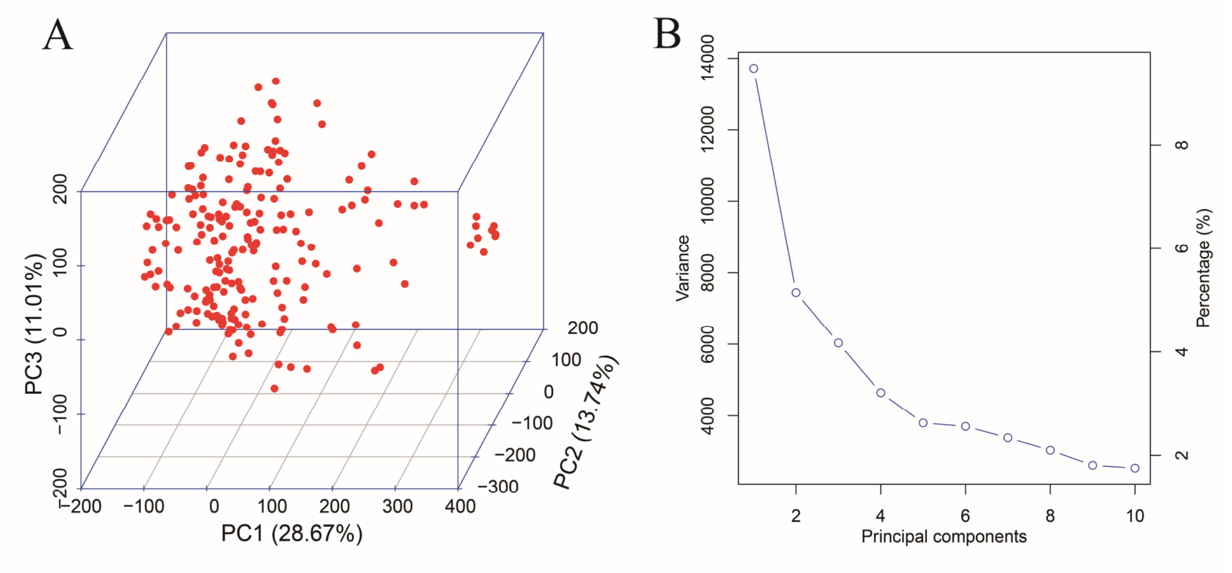

Figure S2. Principal component analysis plots for the association panel comprising 207 wheat cultivars.

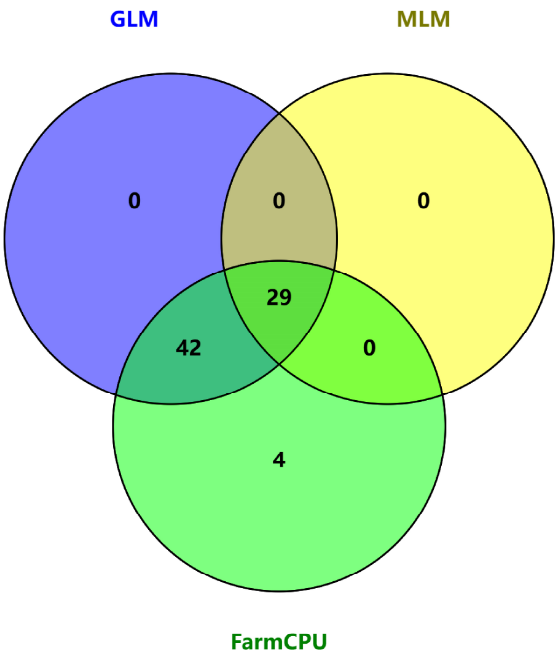

Figure S3. The SNPs significantly associated with Ni content in wheat grains, which were repeatedly identified by the analysis models of GLM, MLM, and FarmCPU.

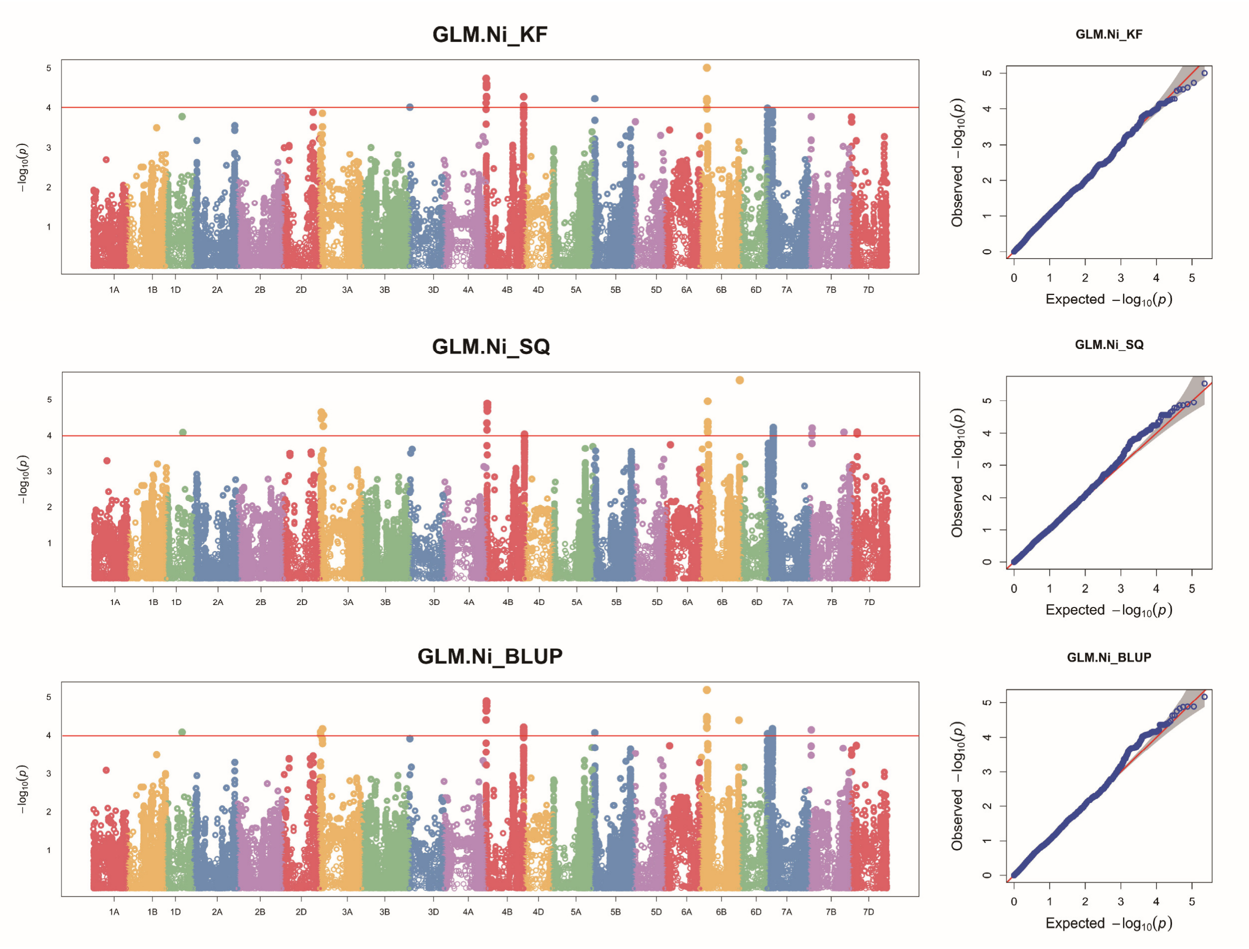

Figure S4. Manhattan and QQ plots for Ni concentrations in wheat grains were generated using the GLM in all the environments. The dashed horizontal line indicates the significance threshold of −log10(P) = 4.0. SNPs above the red dotted line are significantly associated with Ni variation.

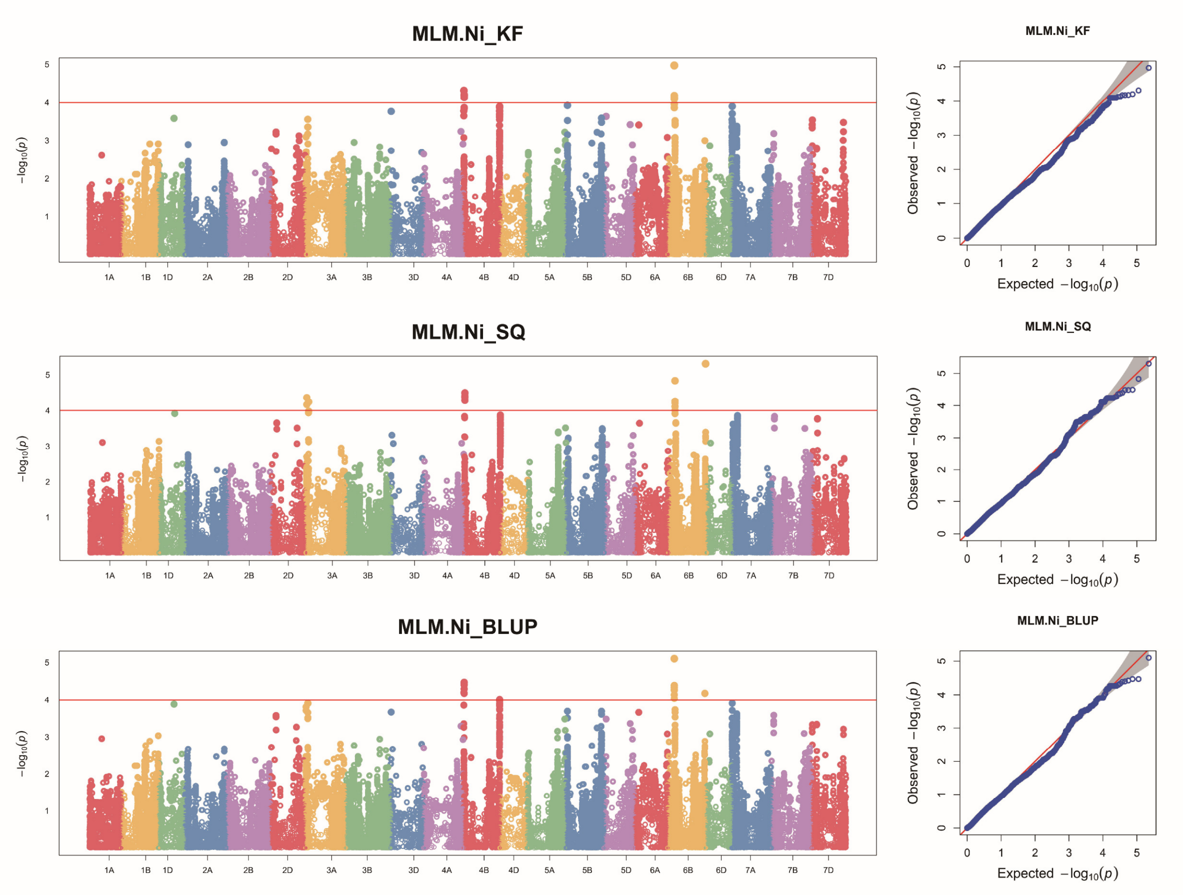

Figure S5. Manhattan and QQ plots for Ni concentrations in wheat grains were generated using the MLM in all the environments. The dashed horizontal line indicates the significance threshold of −log10(P) = 4.0. SNPs above the red dotted line are significantly associated with Ni variation.

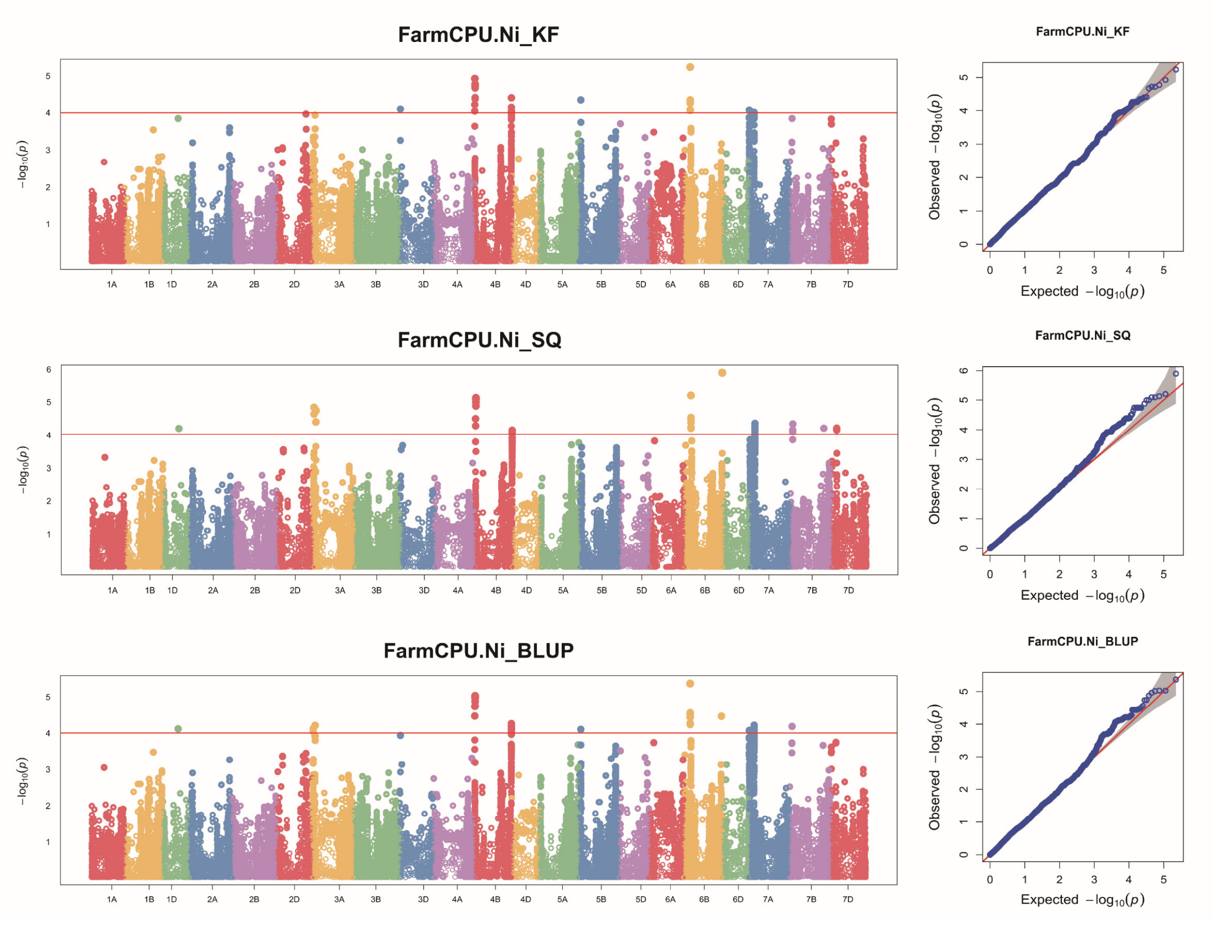

Figure S6. Manhattan and QQ plots for Ni concentrations in wheat grains were generated using the FarmCPU model in all the environments. The dashed horizontal line indicates the significance threshold of −log10(P) = 4.0. SNPs above the red dotted line are significantly associated with Ni variation.

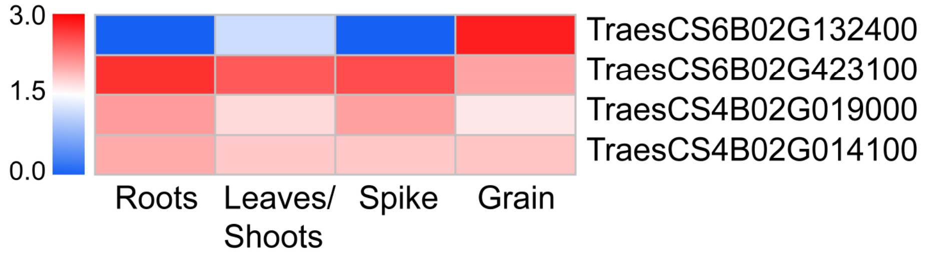

Figure S7. Expression levels of promising candidate genes in different wheat tissues. The heat map was plotted using the transcripts per kilobase million (TPM) values from the gene expression profile public database.

Table S1. The proportion of genetic variation explained by different principal components

PCs	Ratio
PC1	28.67%
PC2	13.74%
PC3	11.01%
PC4	9.44%
PC5	7.37%
PC6	6.98%
PC7	6.49%
PC8	6.10%
PC9	5.43%
PC10	4.76%

Table S2. Marker-trait associations (MTAs) for Ni accumulation in the association population analyzed by the GLM

Environment[1]	SNP marker[2]	Chromosome	Physical Position(bp)	P-value[3]	R^2(%)[4]
KF	AX-111126872	6B	125482475	9.96E-06	13.42
KF	AX-109441323	4B	6642111	1.85E-05	12.80
KF	AX-108959147	4B	11609441	2.49E-05	12.50
KF	AX-109323724	4B	11621019	2.80E-05	12.39
KF	AX-110161827	4B	11593889	2.80E-05	12.39
KF	AX-111152433	4B	11587148	3.06E-05	12.30
KF	AX-111120406	4B	11665725	5.21E-05	11.77
KF	AX-108868218	4B	670377040	5.27E-05	11.76
KF	AX-109856418	4B	11594373	5.56E-05	11.71
KF	AX-110947094	6B	126135342	5.89E-05	11.65
KF	AX-108859035	5B	37265714	5.93E-05	11.65
KF	AX-111602613	6B	126220786	6.52E-05	11.55
KF	AX-109851023	6B	125633588	7.06E-05	11.47
KF	AX-110530193	6B	125612615	7.06E-05	11.47
KF	AX-108726511	6B	125525941	7.06E-05	11.47
KF	AX-110081172	6B	126309780	7.06E-05	11.47
KF	AX-110921034	6B	126134619	7.06E-05	11.47
KF	AX-111001468	6B	125602472	7.06E-05	11.47
KF	AX-109494547	4B	4997767	7.66E-05	11.39
KF	AX-109482775	4B	670432861	8.92E-05	11.25
KF	AX-94458362	3D	15330622	9.87E-05	11.15

SQ	AX-108754008	6B	693584519	2.89E-06	13.39
SQ	AX-111126872	6B	125482475	1.12E-05	11.99
SQ	AX-111120406	4B	11665725	1.29E-05	11.85
SQ	AX-109323724	4B	11621019	1.39E-05	11.78
SQ	AX-110161827	4B	11593889	1.39E-05	11.78
SQ	AX-111152433	4B	11587148	1.66E-05	11.60
SQ	AX-108959147	4B	11609441	1.68E-05	11.59
SQ	AX-109856418	4B	11594373	2.10E-05	11.36
SQ	AX-110424807	3A	15485206	2.29E-05	11.27
SQ	AX-109348794	3A	46735742	2.78E-05	11.07
SQ	AX-94393741	3A	50747385	2.78E-05	11.07
SQ	AX-109533360	3A	46735300	2.78E-05	11.07
SQ	AX-109902302	3A	50382605	2.78E-05	11.07
SQ	AX-112287488	3A	47825432	2.78E-05	11.07
SQ	AX-112289217	3A	49478004	2.78E-05	11.07
SQ	AX-94396571	3A	50382315	2.78E-05	11.07
SQ	AX-108771190	3A	15422968	3.44E-05	10.86
SQ	AX-110947094	6B	126135342	4.23E-05	10.65
SQ	AX-109494547	4B	4997767	4.57E-05	10.58
SQ	AX-111602613	6B	126220786	4.59E-05	10.57
SQ	AX-110464223	3A	50072730	5.54E-05	10.38
SQ	AX-108725296	3A	46835374	5.57E-05	10.38
SQ	AX-108726511	6B	125525941	5.77E-05	10.34

SQ	AX-110081172	6B	126309780	5.77E-05	10.34
SQ	AX-110921034	6B	126134619	5.77E-05	10.34
SQ	AX-111001468	6B	125602472	5.77E-05	10.34
SQ	AX-109851023	6B	125633588	5.77E-05	10.34
SQ	AX-110530193	6B	125612615	5.77E-05	10.34
SQ	AX-110518597	7A	93068668	6.07E-05	10.29
SQ	AX-94490431	7A	93078259	6.29E-05	10.26
SQ	AX-95170264	7B	43882124	6.29E-05	10.26
SQ	AX-109441323	4B	6642111	7.06E-05	10.14
SQ	AX-109897959	7A	92893621	7.63E-05	10.06
SQ	AX-110577183	6B	125485022	7.64E-05	10.06
SQ	AX-109071288	7A	92073127	7.89E-05	10.03
SQ	AX-109599257	7A	92479851	7.89E-05	10.03
SQ	AX-110436030	7A	92483047	7.89E-05	10.03
SQ	AX-110496757	7A	92360773	7.89E-05	10.03
SQ	AX-94906595	7D	90850773	8.16E-05	10.00
SQ	AX-111535365	7B	607313447	8.23E-05	9.99
SQ	AX-109377038	6B	125602039	8.29E-05	9.98
SQ	AX-95204455	1D	291091867	8.35E-05	9.98
SQ	AX-110033252	7A	92471260	8.56E-05	9.95
SQ	AX-94928381	7B	41544017	9.12E-05	9.89
SQ	AX-94761355	7D	90598933	9.12E-05	9.89
SQ	AX-109482775	4B	670432861	9.24E-05	9.87

SQ	AX-109509539	7A	92868303	9.25E-05	9.87
SQ	AX-89511965	7A	93078467	9.71E-05	9.83
SQ	AX-110484935	7A	93055272	9.74E-05	9.82
SQ	AX-110918439	4B	670399775	9.81E-05	9.82
BLUP	AX-111126872	6B	125482475	6.64E-06	13.12
BLUP	AX-109323724	4B	11621019	1.30E-05	12.44
BLUP	AX-110161827	4B	11593889	1.30E-05	12.44
BLUP	AX-108959147	4B	11609441	1.34E-05	12.41
BLUP	AX-111152433	4B	11587148	1.49E-05	12.30
BLUP	AX-111120406	4B	11665725	1.77E-05	12.13
BLUP	AX-109856418	4B	11594373	2.32E-05	11.86
BLUP	AX-109441323	4B	6642111	2.35E-05	11.84
BLUP	AX-110947094	6B	126135342	3.38E-05	11.48
BLUP	AX-111602613	6B	126220786	3.72E-05	11.38
BLUP	AX-109494547	4B	4997767	4.06E-05	11.30
BLUP	AX-108754008	6B	693584519	4.11E-05	11.29
BLUP	AX-108726511	6B	125525941	4.35E-05	11.23
BLUP	AX-110081172	6B	126309780	4.35E-05	11.23
BLUP	AX-110921034	6B	126134619	4.35E-05	11.23
BLUP	AX-111001468	6B	125602472	4.35E-05	11.23
BLUP	AX-109851023	6B	125633588	4.35E-05	11.23
BLUP	AX-110530193	6B	125612615	4.35E-05	11.23
BLUP	AX-110577183	6B	125485022	6.09E-05	10.89

BLUP	AX-109482775	4B	670432861	6.25E-05	10.87
BLUP	AX-109377038	6B	125602039	6.58E-05	10.82
BLUP	AX-110518597	7A	93068668	6.84E-05	10.78
BLUP	AX-109533360	3A	46735300	6.92E-05	10.77
BLUP	AX-109902302	3A	50382605	6.92E-05	10.77
BLUP	AX-112287488	3A	47825432	6.92E-05	10.77
BLUP	AX-112289217	3A	49478004	6.92E-05	10.77
BLUP	AX-94396571	3A	50382315	6.92E-05	10.77
BLUP	AX-109348794	3A	46735742	6.92E-05	10.77
BLUP	AX-94393741	3A	50747385	6.92E-05	10.77
BLUP	AX-110918439	4B	670399775	7.22E-05	10.73
BLUP	AX-94490431	7A	93078259	7.34E-05	10.71
BLUP	AX-95170264	7B	43882124	7.34E-05	10.71
BLUP	AX-110484935	7A	93055272	7.69E-05	10.66
BLUP	AX-108868218	4B	670377040	7.76E-05	10.66
BLUP	AX-89511965	7A	93078467	7.86E-05	10.64
BLUP	AX-109301093	7A	93130503	8.13E-05	10.61
BLUP	AX-109507986	7A	93055918	8.24E-05	10.60
BLUP	AX-109831753	7A	93061650	8.24E-05	10.60
BLUP	AX-110362283	7A	93134998	8.24E-05	10.60
BLUP	AX-110424807	3A	15485206	8.28E-05	10.59
BLUP	AX-95204455	1D	291091867	8.42E-05	10.58
BLUP	AX-111457172	7A	93054845	8.46E-05	10.57

BLUP	AX-111550569	7A	93077706	8.46E-05	10.57
BLUP	AX-109982012	7A	93077291	8.46E-05	10.57
BLUP	AX-109301896	4B	670342450	8.52E-05	10.56
BLUP	AX-109288761	4B	670433105	8.52E-05	10.56
BLUP	AX-110392767	7A	93072454	8.68E-05	10.55
BLUP	AX-108859035	5B	37265714	8.70E-05	10.54
BLUP	AX-110128021	7A	93131508	9.04E-05	10.50
BLUP	AX-95071431	7A	7597513	9.17E-05	10.49
BLUP	AX-110934105	7A	4629077	9.24E-05	10.48
BLUP	AX-108840795	7A	93078145	9.29E-05	10.48
BLUP	AX-109356386	7A	93073586	9.29E-05	10.48
BLUP	AX-110406934	4B	670440098	9.29E-05	10.48
BLUP	AX-109305998	7A	93135273	9.34E-05	10.47
BLUP	AX-109919236	4B	670340875	9.47E-05	10.46
BLUP	AX-108771190	3A	15422968	9.94E-05	10.41

[1] Phenotypic values collected from three locations (KF, 2023 Kaifeng; SQ, 2023 Shangqiu), and the best linear unbiased predictions (BLUP) values calculated from all environments.

[2] Markers were detected at the threshold $-\log_{10}(P)$ equaling 4.0.

[3] P-value of the corresponding significant SNPs calculated by GLM model.

[4] R^2 of Model with significant SNP.

Table S3. Marker-trait associations (MTAs) for Ni accumulation in the association population analyzed by the MLM

Environment[1]	SNP marker[2]	Chromosome	Physical Position(bp)	P-value[3]	R^2(%)[4]
KF	AX-111126872	6B	125482475	1.07E-05	11.29
KF	AX-109441323	4B	6642111	6.63E-05	9.44
KF	AX-108959147	4B	11609441	6.23E-05	9.51
KF	AX-110947094	6B	126135342	6.57E-05	9.45
KF	AX-109323724	4B	11621019	6.63E-05	9.44
KF	AX-110161827	4B	11593889	4.84E-05	9.76
KF	AX-111152433	4B	11587148	7.34E-05	9.34
KF	AX-111602613	6B	126220786	7.43E-05	9.33
KF	AX-108726511	6B	125525941	7.96E-05	9.26
KF	AX-110081172	6B	126309780	7.96E-05	9.26
KF	AX-110921034	6B	126134619	7.96E-05	9.26
KF	AX-111001468	6B	125602472	7.96E-05	9.26
KF	AX-109851023	6B	125633588	7.96E-05	9.26
KF	AX-110530193	6B	125612615	7.96E-05	9.26
SQ	AX-108754008	6B	693584519	4.94E-06	10.58
SQ	AX-111126872	6B	125482475	1.47E-05	9.43
SQ	AX-111120406	4B	11665725	3.23E-05	8.61
SQ	AX-109323724	4B	11621019	3.23E-05	8.61
SQ	AX-110161827	4B	11593889	3.18E-05	8.63

SQ	AX-111152433	4B	11587148	3.96E-05	8.40
SQ	AX-108959147	4B	11609441	4.24E-05	8.33
SQ	AX-110424807	3A	15485206	4.34E-05	8.31
SQ	AX-109856418	4B	11594373	5.24E-05	8.12
SQ	AX-110947094	6B	126135342	5.49E-05	8.07
SQ	AX-109348794	3A	46735742	5.76E-05	8.02
SQ	AX-94393741	3A	50747385	5.76E-05	8.02
SQ	AX-109533360	3A	46735300	5.76E-05	8.02
SQ	AX-109902302	3A	50382605	5.76E-05	8.02
SQ	AX-112287488	3A	47825432	5.76E-05	8.02
SQ	AX-112289217	3A	49478004	5.76E-05	8.02
SQ	AX-94396571	3A	50382315	5.76E-05	8.02
SQ	AX-111602613	6B	126220786	6.13E-05	7.96
SQ	AX-108771190	3A	15422968	6.82E-05	7.85
SQ	AX-108726511	6B	125525941	7.72E-05	7.72
SQ	AX-110081172	6B	126309780	7.72E-05	7.72
SQ	AX-110921034	6B	126134619	7.72E-05	7.72
SQ	AX-111001468	6B	125602472	7.72E-05	7.72
SQ	AX-109851023	6B	125633588	7.72E-05	7.72
SQ	AX-110530193	6B	125612615	7.72E-05	7.72
SQ	AX-110577183	6B	125485022	9.88E-05	7.47
BLUP	AX-111126872	6B	125482475	7.94E-06	10.77

BLUP	AX-109323724	4B	11621019	3.40E-05	9.27
BLUP	AX-110161827	4B	11593889	3.40E-05	9.27
BLUP	AX-108959147	4B	11609441	3.76E-05	9.16
BLUP	AX-111152433	4B	11587148	3.97E-05	9.11
BLUP	AX-110947094	6B	126135342	4.11E-05	9.07
BLUP	AX-111602613	6B	126220786	4.66E-05	8.94
BLUP	AX-111120406	4B	11665725	5.09E-05	8.86
BLUP	AX-108726511	6B	125525941	5.42E-05	8.79
BLUP	AX-110081172	6B	126309780	5.42E-05	8.79
BLUP	AX-110921034	6B	126134619	5.42E-05	8.79
BLUP	AX-111001468	6B	125602472	5.42E-05	8.79
BLUP	AX-109851023	6B	125633588	5.42E-05	8.79
BLUP	AX-110530193	6B	125612615	5.42E-05	8.79
BLUP	AX-109441323	4B	6642111	6.37E-05	8.63
BLUP	AX-109856418	4B	11594373	6.68E-05	8.58
BLUP	AX-108754008	6B	693584519	6.69E-05	8.58
BLUP	AX-110577183	6B	125485022	7.32E-05	8.49
BLUP	AX-109377038	6B	125602039	9.29E-05	8.25
BLUP	AX-109482775	4B	670432861	9.70E-05	8.20

[1] Phenotypic values collected from three locations (KF, 2023 Kaifeng; SQ, 2023 Shangqiu), and the best linear unbiased predictions (BLUP) values calculated from all environments.

[2] Markers were detected at the threshold $-\log_{10}(P)$ equaling 4.0.

[3] P-value of the corresponding significant SNPs calculated by MLM model.

[4] R^2 of Model with significant SNP.

Table S4. Marker-trait associations (MTAs) for Ni accumulation in the association population analyzed by the FarmCPU

Environment[1]	SNP marker[2]	Chromosome	Physical Position(bp)	P-value[3]
KF	AX-111126872	6B	125482475	5.75E-06
KF	AX-109441323	4B	6642111	1.18E-05
KF	AX-108959147	4B	11609441	1.66E-05
KF	AX-109323724	4B	11621019	1.90E-05
KF	AX-110161827	4B	11593889	1.90E-05
KF	AX-111152433	4B	11587148	2.10E-05
KF	AX-111120406	4B	11665725	3.85E-05
KF	AX-108868218	4B	670377040	3.89E-05
KF	AX-109856418	4B	11594373	4.14E-05
KF	AX-110947094	6B	126135342	4.42E-05
KF	AX-108859035	5B	37265714	4.45E-05
KF	AX-111602613	6B	126220786	4.95E-05
KF	AX-108726511	6B	125525941	5.42E-05
KF	AX-110081172	6B	126309780	5.42E-05
KF	AX-110921034	6B	126134619	5.42E-05
KF	AX-111001468	6B	125602472	5.42E-05
KF	AX-109851023	6B	125633588	5.42E-05
KF	AX-110530193	6B	125612615	5.42E-05
KF	AX-109494547	4B	4997767	5.93E-05

KF	AX-109482775	4B	670432861	7.04E-05
KF	AX-94458362	3D	15330622	7.87E-05
KF	AX-110577183	6B	125485022	8.09E-05
KF	AX-110934105	7A	4629077	8.36E-05
KF	AX-95071431	7A	7597513	8.47E-05
KF	AX-109377038	6B	125602039	8.73E-05
KF	AX-110918439	4B	670399775	8.89E-05
KF	AX-110911144	4B	4713192	8.99E-05
KF	AX-110406934	4B	670440098	9.27E-05
KF	AX-109301093	7A	93130503	9.76E-05
KF	AX-109305998	7A	93135273	9.91E-05
SQ	AX-108754008	6B	693584519	1.27E-06
SQ	AX-111126872	6B	125482475	6.34E-06
SQ	AX-111120406	4B	11665725	7.47E-06
SQ	AX-109323724	4B	11621019	8.14E-06
SQ	AX-110161827	4B	11593889	8.14E-06
SQ	AX-111152433	4B	11587148	9.98E-06
SQ	AX-108959147	4B	11609441	1.01E-05
SQ	AX-109856418	4B	11594373	1.31E-05
SQ	AX-110424807	3A	15485206	1.45E-05
SQ	AX-109533360	3A	46735300	1.81E-05
SQ	AX-109902302	3A	50382605	1.81E-05

SQ	AX-112287488	3A	47825432	1.81E-05
SQ	AX-112289217	3A	49478004	1.81E-05
SQ	AX-94396571	3A	50382315	1.81E-05
SQ	AX-109348794	3A	46735742	1.81E-05
SQ	AX-94393741	3A	50747385	1.81E-05
SQ	AX-108771190	3A	15422968	2.31E-05
SQ	AX-110947094	6B	126135342	2.92E-05
SQ	AX-109494547	4B	4997767	3.20E-05
SQ	AX-111602613	6B	126220786	3.21E-05
SQ	AX-110464223	3A	50072730	3.98E-05
SQ	AX-108725296	3A	46835374	4.00E-05
SQ	AX-108726511	6B	125525941	4.16E-05
SQ	AX-110081172	6B	126309780	4.16E-05
SQ	AX-110921034	6B	126134619	4.16E-05
SQ	AX-111001468	6B	125602472	4.16E-05
SQ	AX-109851023	6B	125633588	4.16E-05
SQ	AX-110530193	6B	125612615	4.16E-05
SQ	AX-110518597	7A	93068668	4.40E-05
SQ	AX-94490431	7A	93078259	4.59E-05
SQ	AX-95170264	7B	43882124	4.59E-05
SQ	AX-109441323	4B	6642111	5.23E-05
SQ	AX-109897959	7A	92893621	5.70E-05

SQ	AX-110577183	6B	125485022	5.71E-05
SQ	AX-109071288	7A	92073127	5.92E-05
SQ	AX-109599257	7A	92479851	5.92E-05
SQ	AX-110436030	7A	92483047	5.92E-05
SQ	AX-110496757	7A	92360773	5.92E-05
SQ	AX-94906595	7D	90850773	6.15E-05
SQ	AX-111535365	7B	607313447	6.21E-05
SQ	AX-109377038	6B	125602039	6.26E-05
SQ	AX-95204455	1D	291091867	6.31E-05
SQ	AX-110033252	7A	92471260	6.49E-05
SQ	AX-94928381	7B	41544017	6.96E-05
SQ	AX-94761355	7D	90598933	6.96E-05
SQ	AX-109482775	4B	670432861	7.07E-05
SQ	AX-109509539	7A	92868303	7.08E-05
SQ	AX-89511965	7A	93078467	7.47E-05
SQ	AX-110484935	7A	93055272	7.50E-05
SQ	AX-110918439	4B	670399775	7.56E-05
SQ	AX-109507986	7A	93055918	7.82E-05
SQ	AX-109831753	7A	93061650	7.82E-05
SQ	AX-110362283	7A	93134998	7.82E-05
SQ	AX-94695736	7B	41543943	7.87E-05
SQ	AX-109857318	7A	92479814	8.20E-05

SQ	AX-109919236	4B	670340875	8.28E-05
SQ	AX-111457172	7A	93054845	8.39E-05
SQ	AX-111550569	7A	93077706	8.39E-05
SQ	AX-109982012	7A	93077291	8.39E-05
SQ	AX-109301896	4B	670342450	8.45E-05
SQ	AX-109288761	4B	670433105	8.45E-05
SQ	AX-110128021	7A	93131508	8.60E-05
SQ	AX-110392767	7A	93072454	8.88E-05
SQ	AX-109301093	7A	93130503	8.92E-05
SQ	AX-108840795	7A	93078145	9.23E-05
SQ	AX-109356386	7A	93073586	9.23E-05
SQ	AX-109283716	7A	92392963	9.49E-05
BLUP	AX-111126872	6B	125482475	4.29E-06
BLUP	AX-109323724	4B	11621019	9.29E-06
BLUP	AX-110161827	4B	11593889	9.29E-06
BLUP	AX-108959147	4B	11609441	9.62E-06
BLUP	AX-111152433	4B	11587148	1.09E-05
BLUP	AX-111120406	4B	11665725	1.32E-05
BLUP	AX-109856418	4B	11594373	1.80E-05
BLUP	AX-109441323	4B	6642111	1.82E-05
BLUP	AX-110947094	6B	126135342	2.75E-05
BLUP	AX-111602613	6B	126220786	3.06E-05

BLUP	AX-109494547	4B	4997767	3.37E-05
BLUP	AX-108754008	6B	693584519	3.42E-05
BLUP	AX-108726511	6B	125525941	3.65E-05
BLUP	AX-110081172	6B	126309780	3.65E-05
BLUP	AX-110921034	6B	126134619	3.65E-05
BLUP	AX-111001468	6B	125602472	3.65E-05
BLUP	AX-109851023	6B	125633588	3.65E-05
BLUP	AX-110530193	6B	125612615	3.65E-05
BLUP	AX-110577183	6B	125485022	5.30E-05
BLUP	AX-109482775	4B	670432861	5.46E-05
BLUP	AX-109377038	6B	125602039	5.78E-05
BLUP	AX-110518597	7A	93068668	6.04E-05
BLUP	AX-109533360	3A	46735300	6.12E-05
BLUP	AX-109902302	3A	50382605	6.12E-05
BLUP	AX-112287488	3A	47825432	6.12E-05
BLUP	AX-112289217	3A	49478004	6.12E-05
BLUP	AX-94396571	3A	50382315	6.12E-05
BLUP	AX-109348794	3A	46735742	6.12E-05
BLUP	AX-94393741	3A	50747385	6.12E-05
BLUP	AX-110918439	4B	670399775	6.40E-05
BLUP	AX-94490431	7A	93078259	6.52E-05
BLUP	AX-95170264	7B	43882124	6.52E-05

BLUP	AX-110484935	7A	93055272	6.87E-05
BLUP	AX-108868218	4B	670377040	6.93E-05
BLUP	AX-89511965	7A	93078467	7.03E-05
BLUP	AX-109301093	7A	93130503	7.30E-05
BLUP	AX-109507986	7A	93055918	7.41E-05
BLUP	AX-109831753	7A	93061650	7.41E-05
BLUP	AX-110362283	7A	93134998	7.41E-05
BLUP	AX-110424807	3A	15485206	7.45E-05
BLUP	AX-95204455	1D	291091867	7.59E-05
BLUP	AX-111457172	7A	93054845	7.63E-05
BLUP	AX-111550569	7A	93077706	7.63E-05
BLUP	AX-109982012	7A	93077291	7.63E-05
BLUP	AX-109301896	4B	670342450	7.69E-05
BLUP	AX-109288761	4B	670433105	7.69E-05
BLUP	AX-110392767	7A	93072454	7.84E-05
BLUP	AX-108859035	5B	37265714	7.87E-05
BLUP	AX-110128021	7A	93131508	8.21E-05
BLUP	AX-95071431	7A	7597513	8.33E-05
BLUP	AX-110934105	7A	4629077	8.41E-05
BLUP	AX-108840795	7A	93078145	8.45E-05
BLUP	AX-109356386	7A	93073586	8.45E-05
BLUP	AX-110406934	4B	670440098	8.46E-05

BLUP	AX-109305998	7A	93135273	8.50E-05
BLUP	AX-109919236	4B	670340875	8.63E-05
BLUP	AX-108771190	3A	15422968	9.11E-05
BLUP	AX-110033252	7A	92471260	9.61E-05

[1] Phenotypic values collected from three locations (KF, 2023 Kaifeng; SQ, 2023 Shangqiu), and the best linear unbiased predictions (BLUP) values calculated from all environments.

[2] Markers were detected at the threshold $-\log_{10}(P)$ equaling 4.0.

[3] P-value of the corresponding significant SNPs calculated by FarmCPU model.

.

TableS5. Differentially expressed genes annotation in all the integrated loci

Peak SNP	Candidate genes[1]	Expression level in tissues (TPM)[2]				Annotation[3]
		Roots	Leaves	Spike	Grain	
AX-110161827	TraesCS4B02G017500	0.00	3.82	0.00	9.46	Globulin 1
	TraesCS4B02G017600	0.00	0.00	0.00	8.25	Globulin-1
	TraesCS4B02G020300	9.19	9.44	9.43	7.88	RNA-binding protein
	TraesCS4B02G014100	2.99	2.53	2.55	2.60	Membrin
	TraesCS4B02G020000	4.67	3.03	3.76	2.44	Aminopeptidase
	TraesCS4B02G019000	3.25	2.29	3.17	2.12	Transmembrane protein 56
	TraesCS4B02G015300	1.13	0.00	0.59	0.68	Non specific phospholipase C
	TraesCS4B02G010400	1.57	5.40	4.13	0.57	Aldo-keto reductase, putative
	TraesCS4B02G021400	0.21	1.01	0.99	0.17	F-box domain containing protein, expressed
	TraesCS4B02G019800	3.19	0.69	2.58	0.15	Deoxyuridine 5'-triphosphate nucleotidohydrolase
	TraesCS4B02G018900	1.73	2.26	1.22	0.04	Regulatory protein NPR1
AX-111126872	TraesCS6B02G132400	0.00	1.25	0.00	6.07	Glycine rich protein
	TraesCS6B02G129800	6.78	5.91	6.29	4.96	Calreticulin/calnexin
	TraesCS6B02G130600	3.51	2.17	3.24	4.31	CTC-interacting domain 7
	TraesCS6B02G129700	3.39	2.65	3.21	3.14	AT5G11810-like protein

	TraesCS6B02G130900	2.42	2.90	1.88	2.47	Aspartyl/glutamyl-tRNA(Asn/Gln) amidotransferase subunit B
	TraesCS6B02G131100	3.92	4.21	3.14	2.24	Prolyl endopeptidase
	TraesCS6B02G130700	0.23	1.73	1.07	1.82	5'-AMP-activated protein kinase subunit beta-1
	TraesCS6B02G129600	2.84	1.33	2.06	1.57	DNA polymerase V
	TraesCS6B02G128600	0.00	0.00	0.00	1.48	BURP domain protein RD22
	TraesCS6B02G131000	1.77	1.36	1.20	1.47	Laminin subunit gamma-1
	TraesCS6B02G127800	0.00	0.00	0.00	0.45	Mitogen-activated protein kinase
	TraesCS6B02G126900	0.53	0.04	1.35	0.25	Kinetochore protein nuf2, putative
	TraesCS6B02G126300	0.44	0.00	0.09	0.19	evolutionarily conserved C-terminal region 6
	TraesCS6B02G133600	0.00	0.00	0.54	0.12	F-box family protein
	TraesCS6B02G127100	2.68	1.86	1.73	0.12	DUF1645 family protein
	TraesCS6B02G127300	0.30	1.43	1.31	0.08	Succinate dehydrogenase [ubiquinone] flavoprotein subunit, mitochondrial
AX-108754008	TraesCS6B02G424800	8.10	5.34	6.34	5.86	50S ribosomal protein L14
	TraesCS6B02G423100	5.53	4.56	4.89	3.12	Glutathione reductase
	TraesCS6B02G419500	3.20	1.60	3.06	2.58	Octicosapeptide/Phox/Bem1p domain-containing protein
	TraesCS6B02G417700	3.82	2.69	3.21	2.39	Ankyrin repeat protein-like
	TraesCS6B02G423500	3.31	1.99	2.82	2.31	F-box protein PP2

	TraesCS6B02G423200	2.03	0.98	1.08	0.12	F-box protein PP2

[1] The expression levels of the candidate gene are significantly different among different haplotype groups.

[2] Expression level of genes in different tissues were sieved out from the Wheat Expression Browser (http://www.wheat-expression.com), TMP values was the transcripts per kilobase of exon model per million mapped reads.

[3] Gene function annotation of the reference genome IWGSC - RefSeq - v1.1.

TableS6. The expression of candidate genes among different haplotype groups associated with significant loci

Peak SNP	Candidate genes	Allele type[1]		P-value
		Superior	Inferior	
AX-110161827	TraesCS4B02G017500	1497.71±883.74	935.44±437.36	1.08E-04
	TraesCS4B02G021400	1.55±0.56	1.97±0.83	1.46E-04
	TraesCS4B02G020000	10.75±3.44	8.57±2.60	1.93E-04
	TraesCS4B02G018900	4.27±1.32	5.06±1.64	1.38E-03
	TraesCS4B02G017600	1022.07±571.46	706.56±648.79	2.39E-03
	TraesCS4B02G019000	3.84±1.24	4.50±1.54	4.17E-03
	TraesCS4B02G019800	2.25±1.74	1.44±1.29	5.53E-03
	TraesCS4B02G015300	18.32±10.18	14.17±8.36	1.67E-02
	TraesCS4B02G010400	1.36±0.84	1.03±0.78	2.30E-02
	TraesCS4B02G020300	178.47±85.32	213.70±121.61	3.23E-02

	TraesCS4B02G014100	6.03±1.33	5.82±1.62	3.89E-01
AX-111126872	TraesCS6B02G128600	5.23±3.09	0.38±1.10	4.73E-11
	TraesCS6B02G129600	3.07±0.99	4.29±1.22	7.20E-07
	TraesCS6B02G127100	8.25±8.94	20.37±24.71	9.84E-06
	TraesCS6B02G127800	1.24±1.64	2.92±1.93	2.88E-05
	TraesCS6B02G131000	3.33±1.39	2.10±0.70	1.29E-04
	TraesCS6B02G130600	13.39±6.59	7.66±3.21	1.66E-04
	TraesCS6B02G129700	9.88±3.44	12.81±2.14	2.47E-04
	TraesCS6B02G129800	32.28±13.06	21.62±5.43	3.82E-04
	TraesCS6B02G130900	2.39±2.51	0.36±0.93	4.29E-04
	TraesCS6B02G126900	1.48±0.49	1.17±0.38	5.90E-03
	TraesCS6B02G126300	1.44±0.69	1.012±0.45	6.72E-03
	TraesCS6B02G127300	1.43±0.55	1.14±0.53	2.67E-02

	TraesCS6B02G132400	104.51±112.56	158.91±97.67	3.89E-02
	TraesCS6B02G133600	1.53±0.56	1.27±0.50	4.38E-02
	TraesCS6B02G130700	3.91±2.99	2.54±2.35	4.89E-02
	TraesCS6B02G131100	8.52±2.58	7.35±1.51	4.90E-02
AX-108754008	TraesCS6B02G423200	0.65±0.45	1.28±1.03	1.23E-04
	TraesCS6B02G419500	4.77±1.81	6.97±3.70	5.27E-04
	TraesCS6B02G423500	8.40±3.59	12.49±4.46	6.47E-04
	TraesCS6B02G424800	25.78±67.57	83.74±97.30	1.04E-02
	TraesCS6B02G417700	5.38±1.23	6.37±1.29	1.36E-02
	TraesCS6B02G423100	12.46±2.73	14.70±5.77	1.94E-02

[1] The expression of candidate genes among different haplotype groups associated with significant loci are available in Genome Sequence Archive (https://bigd.big.ac.cn/gsa/browse/CRA004223).

TableS7. Raw data of the expression analysis of candidate genes in the groups with high and low Ni contents

Group	Accession ID.	100 Grain weight (g)			Ni concentrations (μg/kg)			Candidate genes expression (FPKM)[1]			
		KF	SQ	BLUP	KF	SQ	BLUP	TraesCS6B02G 132400	TraesCS6B02G 423100	TraesCS4B02G 019000	TraesCS4B02G 014100
Group I	L019	5.11	5.05	4.99	56.94	50.43	55.63	88.30	12.08	5.04	7.10
	L022	3.46	4.15	3.61	47.44	48.79	50.34	20.57	10.84	4.00	7.88
	L064	5.05	5.21	5.07	50.85	55.56	55.17	45.06	11.16	3.25	5.93
	L068	4.86	5.60	5.26	43.20	62.59	54.88	10.70	9.18	3.68	5.98
	L071	4.38	5.26	4.89	23.46	43.13	36.28	24.97	9.33	4.02	5.81
	L089	5.28	5.30	5.19	43.82	63.60	55.65	76.66	11.28	4.49	6.07
	L106	4.71	3.47	3.98	46.36	68.10	58.99	83.63	11.22	2.45	6.95
	L119	5.26	5.30	5.39	51.36	63.91	59.38	113.95	14.74	3.42	5.00
	L122	4.75	4.74	4.82	57.11	58.13	59.36	38.51	11.14	4.39	5.81

	L138	4.24	4.03	4.06	59.31	49.87	56.48	85.23	13.34	4.36	5.70
	L143	6.72	6.50	6.05	41.76	68.82	57.15	115.38	9.14	3.97	5.68
	L145	5.28	5.07	5.01	43.32	60.77	54.08	13.00	13.60	3.72	3.73
	L177	3.97	4.70	4.45	37.98	58.65	50.53	35.80	8.05	4.66	4.53
	L184	5.04	4.98	4.96	53.18	58.24	57.55	131.22	11.37	3.82	6.60
	L191	4.87	3.89	4.49	51.46	60.58	57.84	0.71	11.59	2.27	9.06
Group II	L043	2.99	2.83	3.03	108.36	139.50	122.27	173.74	13.70	4.94	5.10
	L052	3.93	3.37	3.98	151.80	122.18	134.67	120.68	11.90	7.61	5.02
	L075	4.78	5.22	4.96	145.88	114.21	128.08	98.98	12.35	4.23	5.59
	L076	5.17	5.70	5.34	154.85	125.17	137.54	54.29	12.23	3.32	6.61
	L078	4.94	4.78	5.02	142.33	128.44	133.15	27.54	12.83	5.15	7.03
	L094	4.51	3.93	4.10	152.54	135.05	141.13	193.83	16.69	4.19	5.61
	L135	5.40	5.22	5.15	165.56	172.83	165.23	60.71	12.87	3.53	7.04

L152	5.19	4.90	4.81	128.87	170.19	146.57	44.02	13.30	3.45	5.39
L157	4.08	4.33	4.15	294.05	287.27	280.47	138.06	28.99	5.03	2.99
L167	3.23	3.14	3.39	109.54	150.79	128.19	341.24	14.44	3.90	3.21
L190	5.05	5.33	5.18	223.59	216.52	213.48	81.34	11.86	4.79	5.34
L198	4.76	4.83	4.80	206.28	214.09	204.12	61.96	12.41	3.34	5.55
L202	4.87	4.80	4.90	185.83	175.01	175.87	60.83	13.70	7.97	4.35
L204	4.46	5.03	4.84	193.75	175.72	179.97	161.99	11.56	4.70	3.41
L206	5.16	5.06	5.14	236.89	198.38	211.19	338.26	13.15	4.30	5.76

[1] The expression of candidate genes among different groups with high and low Ni contents are available in Genome Sequence Archive (https://bigd.big.ac.cn/gsa/browse/CRA004223).

TableS8. Analysis of the differences in 100-grain weight and Ni content between the groups with high and low Ni concentrations

Traits	Environments	Mean ± SD		P-value[1]
		Group I	Group II	
Ni Concentration (μg/kg)	KF	47.17±9.05	173.34±50.76	1.08E-07
	SQ	58.08±7.35	168.36±46.56	2.18E-07
	BLUP	54.62±5.78	166.80±44.55	1.06E-07
100 Grain weight (g)	KF	4.87±0.73	4.57±0.72	2.71E-01
	SQ	4.88±0.76	4.57±0.86	2.95E-01
	BLUP	4.82±0.62	4.59±0.70	3.44E-01

[1] The P-value data are derived from the ANOVA. A P-value less than 0.05 indicates a significant difference, and a P-value less than 0.01 indicates an extremely significant difference.

TableS9. Analysis of the expression of candidate genes in the groups with high and low Ni contents

Candidate genes	Gene Expression (FPKM)		P-value[1]
	Group I	Group II	
TraesCS6B02G132400	58.91±42.69	130.50±98.68	1.80E-02
TraesCS6B02G423100	11.20±1.81	14.13±4.30	2.50E-02
TraesCS4B02G019000	3.84±0.76	4.70±1.40	4.90E-02
TraesCS4B02G014100	6.12±1.30	5.20±1.26	6.0E-02

[1] The P-value data are derived from the ANOVA. A P-value less than 0.05 indicates a significant difference, and a P-value less than 0.01 indicates an extremely significant difference.

Table S10. The average phenotypic values for Ni accumulation across different environments in 207 wheat accessions, along with their BLUP estimates

Accession ID.	Cultivar name	KF (μg/kg)	SQ (μg/kg)	BLUP
L001	Changwu 135	53.20	58.11	57.50
L002	Shan 229	107.05	104.07	104.85
L003	Xiaoyan 6	60.80	66.74	65.20
L004	Lantian 10	53.48	53.42	55.41
L005	Luohan 3	57.69	54.84	58.08
L006	Jing 411	70.40	80.34	76.20
L007	Ningdong 1	91.65	105.96	98.44
L008	Mianyang 11	60.80	68.52	66.04
L009	Taishan 5	67.11	84.40	76.57
L010	Xinmai 13	66.62	75.26	72.00
L011	Xinmai 18	78.32	76.64	78.21
L012	Zhoumai 16	79.12	73.57	77.13
L013	Yuanfeng 139	57.20	63.61	62.00
L014	Fengchan 3	95.96	81.02	88.65
L015	Zhongyu 8	97.11	79.08	88.28
L016	Bainong 160	68.56	62.07	66.66
L017	Luomai 21	59.90	97.52	79.37
L018	Yanzhan 4110	57.56	81.00	70.43
L019	Gan 6172	56.94	50.43	55.63
L020	Huaimai 21	86.25	78.38	82.79
L021	Mianyang 26	81.85	95.91	89.02
L022	Lunxuan 715	47.44	48.79	50.34
L023	Youpi 1	38.90	45.74	44.84
L024	Xifeng 9	40.92	56.89	51.09
L025	Nongda 198	79.16	66.29	73.69
L026	Kedong 81	64.43	98.37	81.92
L027	Linfeng 10	71.64	83.89	78.48

L028	Fengkang 5	59.83	62.16	62.56
L029	Changfeng 1	88.24	97.13	92.63
L030	Jingdong 1	114.97	152.83	131.74
L031	Jinmai 21	72.66	82.70	78.39
L032	Jimai 23	120.06	166.39	140.58
L033	Hanxuan 10	68.08	67.69	69.10
L034	Xianshixinmai	68.85	75.13	73.00
L035	Duckbill wheat	79.33	92.10	86.02
L036	Bima 1	74.66	88.52	82.10
L037	Aimengniu	98.51	113.81	105.42
L038	Wenmai 6	78.16	90.37	84.64
L039	Zhameng wheat	131.61	101.90	115.47
L040	Dayuhua	98.90	110.86	104.20
L041	Chushanbao	106.74	90.28	98.16
L042	Dalibanmang	111.23	109.40	109.36
L043	Baizhameng	108.36	139.50	122.27
L044	Ganmai 8	90.65	114.13	101.84
L045	Yunmai 34	94.40	103.77	98.70
L046	Wuyi wheat	100.86	107.23	103.41
L047	Nanda 2419	76.30	96.25	86.55
L048	Nonglin 10	102.29	139.15	119.23
L049	Hanyang wheat	76.80	73.69	76.08
L050	Lovelin 10	87.10	99.50	93.22
L051	Aifeng 3	78.29	82.60	81.02
L052	Yunhan 618	151.80	122.18	134.67
L053	Yannong 21	114.64	103.50	108.18
L054	Jinmai 47	128.90	146.62	135.40
L055	Changwu 58	84.71	93.82	89.39
L056	Zhenghan 1	67.92	77.54	73.70
L057	Chang 6878	68.15	74.93	72.57
L058	Luohan 1	94.66	96.34	95.30

L059	Luohan 6	55.73	56.00	57.70
L060	Luohan 11	74.47	139.35	106.13
L061	Puxing 5	54.40	60.69	59.29
L062	Ruiquan 24	90.92	108.81	99.45
L063	Dehongfu 2	77.30	99.95	88.78
L064	Zhongmai 895	50.85	55.56	55.17
L065	Huaimai 35	79.19	90.77	85.32
L066	Longping 518	61.54	78.49	71.12
L067	Huaimai 30	72.79	83.35	78.77
L068	Zhongyuan 6	43.20	62.59	54.88
L069	Pingan 8	63.01	76.42	70.84
L070	Bainong 207	60.25	89.11	75.55
L071	Cun 1	23.46	43.13	36.28
L072	Zhoumai 26	52.75	76.26	65.89
L073	Luomai 18	56.56	63.61	61.70
L074	Baofeng 10-82	86.97	79.98	83.89
L075	Su 553	145.88	114.21	128.08
L076	09N37	154.85	125.17	137.54
L077	Guinong 17	106.79	110.02	107.55
L078	Hengguan 35	142.33	128.44	133.15
L079	Yumai 18	119.84	92.11	105.24
L080	Yumai 13	85.91	77.46	82.19
L081	Yumai 47	59.99	76.77	69.57
L082	Zhengmai 004	95.88	88.74	92.27
L083	Yumai 50	82.10	97.46	89.87
L084	Taikong 6	58.93	62.04	62.08
L085	Huapei 5	65.05	66.15	66.93
L086	Hua 9987	63.75	91.85	78.51
L087	Yujiao 5	51.41	76.00	65.13
L088	Yunong 416	113.91	93.68	103.17
L089	Bainong 64	43.82	63.60	55.65

L090	Zhoumai 9	80.95	78.69	80.42
L091	Zhoumai 13	63.58	65.71	66.03
L092	Zhoumai 8425B	62.70	91.02	77.62
L093	Kaimai 21	54.02	68.26	62.70
L094	Luozhen 1	152.54	135.05	141.13
L095	Neixiang 188	71.51	75.12	74.25
L096	Aiyou 26-2	51.94	56.38	56.08
L097	Changgeheimai	61.03	90.32	76.49
L098	Lvmai 1	76.76	95.72	86.52
L099	Subeimai 1	67.85	99.36	84.01
L100	Jinan 17	72.51	98.26	85.70
L101	Jimai 20	97.32	84.23	90.82
L102	Shan 225	242.16	187.21	208.39
L103	Xinong 979	84.80	61.02	73.87
L104	Baxter	72.30	88.75	81.09
L105	CD87	46.01	66.33	57.98
L106	Kukri	46.36	68.10	58.99
L107	Faguomai	76.53	92.09	84.69
L108	Belero	101.49	99.23	99.92
L109	Fundulea 900	43.66	69.11	58.19
L110	Tincurrin	61.11	61.48	62.85
L111	H149	63.86	77.55	71.78
L112	Fa B08	66.69	51.68	60.85
L113	Fa B20	83.29	69.21	77.04
L114	Bainong 416	92.07	83.28	87.88
L115	Luo 31	69.85	94.59	82.70
L116	Taihemai 1	73.21	76.24	75.59
L117	Xunmai 35	59.70	84.46	73.08
L118	Luomai 23	61.05	75.08	69.27
L119	Jiaomai 266	51.36	63.91	59.38
L120	Wennong 14	68.30	74.71	72.54

L121	Jimai 22	74.34	71.18	73.72
L122	Ningmai 9	57.11	58.13	59.36
L123	10EW28	63.45	100.26	82.35
L124	10EW137	74.71	95.53	85.45
L125	Zhou 18	56.28	60.08	59.89
L126	04Z38	74.99	87.85	81.94
L127	Yumai 34-9901	54.96	77.51	67.54
L128	Zheng 9023-8	45.26	59.00	54.15
L129	Zheng 103	56.80	83.19	71.10
L130	Calingiri	84.51	74.52	80.14
L131	Zhengmai 366	92.84	83.03	88.12
L132	Zhengmai 7698	85.59	68.28	77.69
L133	Zhoumai 22	72.06	79.03	76.37
L134	Zhoumai 27	84.53	79.08	82.31
L135	Zhoumai 30	165.56	172.83	165.23
L136	Zhoumai 32	81.86	88.77	85.64
L137	Liangxing 66	86.49	72.43	80.08
L138	Emai 25	59.31	49.87	56.48
L139	Ningchun5	64.04	69.84	68.20
L140	Shannong 33	80.71	90.73	86.02
L141	Zhongmai 175	52.75	84.17	69.65
L142	Gaocheng 8901	80.40	115.91	97.82
L143	Shannongtedali 1	41.76	68.82	57.15
L144	Changmai 9	48.39	60.00	56.11
L145	Yangfumai 2	43.32	60.77	54.08
L146	Wanmai 53	60.59	73.89	68.49
L147	Fanmai 8	50.81	66.23	60.21
L148	Yunmai 51	90.93	82.54	86.98
L149	Yunmai 53	53.12	73.03	64.54
L150	Yunmai 47	85.20	97.72	91.47
L151	Xinong 928	56.72	63.31	61.63

L152	Xinmai 26	128.87	170.19	146.57
L153	Wanmai 47	63.06	74.44	69.92
L154	Yangmai 13	96.25	72.28	84.64
L155	Gaoyou 2018	79.87	194.70	134.95
L156	Gaoyou 9415	172.59	123.16	145.00
L157	Shiluan 02-1	294.05	287.27	280.47
L158	Zheng 1005	118.44	96.78	106.79
L159	Zheng 1105	70.67	79.44	75.90
L160	Zheng 1118	65.77	90.65	78.90
L161	Zheng 1289	59.63	63.57	63.14
L162	Zheng 3093	142.05	191.57	162.96
L163	Sanyuehuang	121.37	119.49	118.96
L164	Wuhuatou	86.96	90.56	88.91
L165	Tutoumai	98.48	98.71	98.24
L166	Baisuibai	53.26	99.17	77.00
L167	Qumangmai	109.54	150.79	128.19
L168	Zhengzhou 6	72.19	104.98	88.74
L169	Zhengyin 1	101.99	124.31	112.05
L170	Shengxuan 7	83.18	89.78	86.74
L171	Shuangfengshou	56.61	67.46	63.55
L172	Zhengzhou 7	81.23	87.94	84.94
L173	Wanchangsui	42.98	58.35	52.76
L174	Yuanzhu	73.99	95.04	84.88
L175	Longfumai 4	50.21	81.97	67.40
L176	Yizheng 8165	50.57	80.23	66.74
L177	Taihan 2	37.98	58.65	50.53
L178	Neixiang 182	45.02	63.12	55.99
L179	Chuanmai 50	67.56	54.18	62.45
L180	Chuanmai 46	59.03	80.20	70.74
L181	Chuanmai 44	45.48	47.51	48.81
L182	Chuanmai 107	68.45	93.94	81.73

L183	Jinyang 35	67.03	72.65	70.96
L184	Zhengmai 101	53.18	58.24	57.55
L185	Zhengmai 518	62.31	73.69	69.21
L186	AK 58	57.26	51.47	56.27
L187	Shannong 22	86.64	73.38	80.61
L188	Nongda 211	71.33	78.17	75.61
L189	Shannong 19	218.50	246.30	225.19
L190	Shannong 06-278	223.59	216.52	213.48
L191	Yangmai 18	51.46	60.58	57.84
L192	Ningmai 17	57.05	63.46	61.86
L193	Ningmai 13	51.84	76.92	65.78
L194	Zhenmai 168	209.19	189.69	193.92
L195	Yangmai 12	202.14	191.84	191.59
L196	Yangmai 14	53.82	77.15	66.82
L197	Huamai 5	180.81	165.95	169.19
L198	Yangmai 20	206.28	214.09	204.12
L199	Yangmai 21	220.07	233.71	219.96
L200	Zhengmai 379	245.45	199.23	215.64
L201	Zhengmai 05706	218.19	192.80	199.66
L202	Zhengmai 113	185.83	175.01	175.87
L203	Zhengmai 369	194.57	163.15	174.39
L204	Zhengmai 05871	193.75	175.72	179.97
L205	Xinmai 19	186.04	185.08	180.75
L206	Shannong 26	236.89	198.38	211.19
L207	Shijiazhuang 8	256.84	287.93	263.13

KF and SQ are Kaifeng 2023 and Shangqiu 2023 in Yellow and Huai wheat region, respectively.

The BLUP values calculated from all environments averaged phenotypic values.
